# Influences of service characteristics and older people’s attributes on outcomes from direct payments

**DOI:** 10.1186/s12877-020-01943-8

**Published:** 2021-01-02

**Authors:** Vanessa Davey

**Affiliations:** 1 Research Fellow, Re-FIT Research Group, Parc Sanitari Pere Virgili & Vall d’Hebrón Institute of Research (VHIR), Barcelona, Spain; 2 Formerly at Personal Social Services Research Unit (PSSRU), London School of Economics & Political Science (LSE), London, UK

**Keywords:** Direct payments, Personal budgets, Consumer-directed care, Older people, Social care outcomes

## Abstract

**Background:**

Direct payments (DPs) are cash-payments that eligible individuals can receive to purchase care services by themselves. DPs are central to current social care policy in England, but their advantages remain controversial. This controversy is partly due to their lack of historical visibility: DPs were deployed in stages, bundled with other policy instruments (first individual budgets, then personal budgets), and amidst increasing budgetary constraints. As a result, little unequivocal evidence is available about the effectiveness of DPs as an instrument for older people’s care. This study aims to partially fill that gap using data obtained during an early evaluation of DP’s that took place between 2005 and 07.

**Methods:**

Semi-structured 81 face-to-face interviews with older people (and their proxies) using DPs are analyzed. DPs contribution to outcomes was measured using a standardized utility scale. Data on individual characteristics (dependency, informal support) and received services (types and amount of services) was also gathered. Multiple regression analyses were performed between measured outcome gains and individual and service characteristics. A Poisson log-functional form was selected to account for the low mean and positive skew of outcome gains.

**Results:**

Levels of met need compared very favorably to average social care outcomes in the domains of social participation, control over daily living and safety, and user satisfaction was high. Benefit from DPs was particularly affected by the role and function of unpaid care and availability of recruitment support. The freedom to combine funded care packages with self-funded care enhanced the positive impact of the former. The ability to purchase care that deviated from standardized care inputs improved service benefits. Large discrepancies between total care input and that supported through DPs negatively affected outcomes.

**Conclusions:**

The results offer clarity regarding the benefit derived from receiving DPs. They also clarify contested aspects of the policy such as the influence of unpaid care, types of care received, funding levels and the role of wider support arrangements. Tangible benefits may results from direct payments but those benefits are highly dependent on policy implementation practices. Implementation of DPs should pay special attention to the balance between DP funded care and unpaid care.

**Supplementary Information:**

The online version contains supplementary material available at 10.1186/s12877-020-01943-8.

## Background

**Fig. 1:**
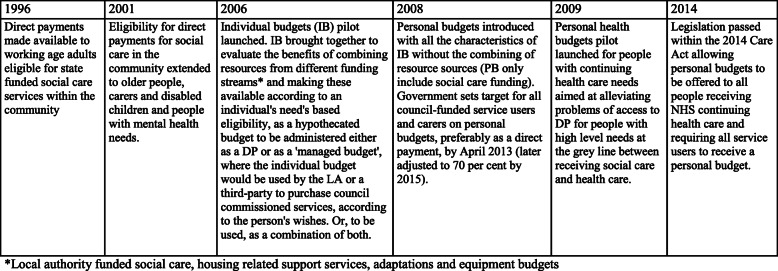
Timeline - direct payments to personal health budgets

During the past decade social care in England has changed substantially as a result of “personalisation” policies (Fig. 1). These changes are subject to significant criticism [1, 2]. Direct Payments (DPs), cost-equivalent cash payments, are now core routes through which individuals eligible for publicly-funded social care can purchase care directly through their “personal” or hypothecated budget (PB). This is a policy drawing on US models of consumer-directed care [3–6], with similarities to recent developments in Australia [7–10] and across Europe [11].

While alternative options are available to those who prefer not to self-manage (Fig. 1), successive governments have attempted to steer implementation of DPs, placing particular emphasis on uptake among older people [12–17]. Despite this, acceptance of DPs for older people has been slow. The government recently referred to the take-up rate for direct payments for older people as “stubbornly low” [18].

Home care^1^ remains the mainstay of support for community-dwelling older people, with only 18% of over 65 s receiving a direct payment, versus 40% of younger people supported because of physical disability [19]. Yet these figures cover a broad range: the top 5% of councils provide DPs to roughly half of all over 65 s receiving care in the community [19] and a similar proportion of councils^2^ now spend more per year on DPs to older people than on homecare [20]. This reflects how personalisation has been used and interpreted differently by different actors [21], surpassing previous patterns of social care variation [22].

The priority given to implement DPs among older people has been questioned. Woolham et al [20, 23] recently challenged the sustained promotion of DPs to older people, stating that current policies fail to recognize that “older people may want different things from personal budgets and direct payments to younger people”. This overlooks the fact that it is often the families of older people who recognize the possible advantages of DPs.

The controversy is fueled by studies in which the suitability of “self-directed care” for older people is questioned. While the initial evidence base was drawn from studies of participant direction in the USA [3, 24], efforts were made to pilot self-management in a local context. The IBSEN study of individual budgets (IBs) (Fig. 1), [25], forerunner to PBs, reported lower psychological wellbeing in older people receiving IBs, compared to either those receiving standard care or to younger IB holders. Even so, no differences were detected in social care need-related outcomes between older and younger participants. Further analysis, excluding proxy responses, found no differences even in psychological wellbeing [26–28].

The conflation of PBs/IBs/DPs, grouped together under the umbrella term “self-directed care”, is problematic in reviewing existing data [25, 29]. IBs and PBs feature major changes in assessment and allocation of publicly funded social care, including introduction of supported self-assessment and notional budgets [30]. It is impossible to discern the impact of actual services received from the impact of how funds are allocated, or how assessment and support planning are handled. PB implementation created significant delays in set-up times for services, increasing service users’ anxieties and impacting on results, particularly among older people [31, 32]. IBs were additionally marred by a “slightly naive attempt to join up funding streams that are very hard to combine” [33].

Existing research is also limited by amalgamation of data on older people that are taking their PB (or IB) as a council-managed budget or a provider-managed budget with those using DPs [25, 27, 31]. PBs managed by local authorities (where the personal budget is “paid to” the council), offer limited participation for recipients in services they receive [34]. Data on provider-managed budgets (also referred to as “Individual Service Funds”) is scarce [34, 35]. Consequently, outcomes data specific to older people in receipt of DPs are extremely limited.

Attempting to address these issues, Woolham et al [36] compared the outcomes of DPs to managed budgets (MBs) among older people. Their findings suggest no significant differences in social care outcomes between the service types, although DP recipients scored higher for process outcomes (timing of care and satisfaction with services). Their findings are in line with official data covering all English councils, available since 2016 as part of the Adult Social Care Survey [35]. Such results suggest a growing mismatch between the Department of Health’s assertion that “direct payments... lead to a higher quality experience for *appropriate* users” and the evidence base [11, 37]. An obvious question is: why there is so much disparity between early qualitative studies [25, 38, 39] and more recent quantitative studies?

Some may argue that DPs were initially offered to those most likely to benefit and as the user base grew those with less to gain were drawn into the pool. Indeed, in early studies of DPs to older people, almost all participants knew about DPs before applying and purposefully requested them [25, 38]. That is clearly different from imposed DP use – an issue raising increasing concern. The incentives for councils to increase uptake of DPs to older people are now such that “practices to promote DPs which work against personalised care” are recognised [35]. This hints at the use of DPs primarily for council interests, particularly “in areas where authority-commissioned care is considered poor quality or where the choice of authority-commissioned providers is very limited …” [34, 35]. Much of this stems from efforts to control costs: between 2006 and 2016 the average unit cost for local authority commissioned home care rose only 21% [40, 41], leaving many providers struggling financially with knock on effects for recruitment and retention of staff. This combines with a switch from cost and volume contracts to ‘framework agreements’ for approved providers which secure potential services at a given cost but do not guarantee service volume to providers [30, 42]. This practice creates such risk to providers that many are opting out of council commissioned care [30]. Reduced supply has led to DPs becoming the last available option.

Concerns have also been raised about the way in which DPs seem to have been pursued as a means of cost-cutting. One London council was cited in the Local Government Association Adult Social Care Efficiency report [43] as having made savings of £0.9 million by, i) switching from in-house domiciliary care to DPs and ii) requiring that any assistance with managing DPs or recruiting care, be paid for by the individual from their allocated funds. This led to a wave of local policy shifts in DP support, from the existing model where support was offered automatically and free at the point of use from schemes contracted by the local authority (an investment roughly equal to 7% of total DP expenditure); to an approach where service users are required to individually purchase assistance from a selection of available providers [44]. This shift overlooked the once heralded role of DP support in improving outcomes [15].

Further changes in the implementation of DPs, have included the introduction of pre-paid card schemes with real time auditing of spending (criticized for reducing flexibility) and online PA recruitment platforms. Such developments have been interpreted as circumventing the “need” for DP support, thereby mitigating its cost [44]. The latter are not unlike the so called ‘Uber-style’ employment management schemes taking hold in Australia under the National Disability Insurance Scheme self-directed care option [45]. There has since been a significant shift in the use of PAs: predominant in the early model of DP use, now only around 1/3rd of direct payments users (all ages) employ one or more PA [46]. The potential problems of hiring a PA are continuously overemphasized [33]. Moreover, conventional homecare agencies, once largely disinterested in targeting DP recipients, now actively do so and have been *encouraged* to diversify to offer PA matching and management services [44]. All these changes to the context in which DPs are being used have gone unrecognized amidst the focus on quantifying whether DPs offer greater benefits than managed budgets (where the local authority organizes care on behalf of the person) for older people.

In the face of so much change in the context in which DPs are provided, there is a pressing need to unpick the, “apparent contradiction between [early] user-level and [recent] authority-level data” [7, 35]. To do so requires exploring how outcomes are influenced by individual characteristics, circumstances and care packages (not just the amount but also *what* is purchased and with what support).

Little data has been collected having this potential. Survey data trades off the benefits of greater sample size with the depth of information collected. It also excludes proxy responses [47], thereby excluding older people who have their DP managed by an appointee, an important subsection of this user group. An exception is the detail contained in 81 face-to-face interviews with older people, undertaken as part of an early Department of Health funded study of DPs to older people immediately prior to the introduction of PBs. These data, newly analysed, give unprecedented depth of view, while their historical nature provides distance from the complex currents in which DPs are now immersed, allowing examination of the possible reasons for the contradiction between (early) user-level and (current) authority-level data.

## Methods

### Recruitment

Older people receiving DPs were recruited from ten councils and interviewed between 2005 and 2007 as part of a wider national evaluation on DPs to older people conducted for the Department of Health, England. Councils were selected to represent a spread of DP take-up rates. The top and lowest performing councils were excluded; the first having already been researched, the latter because of sample size concerns. Participating councils were from the first, second and fourth quartiles for take-up. Selected councils were dotted across the whole of England, split equally between high and low population-density areas.

All older people in receipt of a DP in each council were contacted via a letter, distributed by councils to ensure anonymity. Individuals received information on the study and a freepost envelope to return if they wanted to participate. Eight service users were sought per council, roughly half the national average of older people receiving DPs per council in 2007 [48]. In areas with more positive responses than required, individuals were chosen to give the widest geographical spread within each council. Recipients were chosen irrespective of whether or not they had an unpaid carer.

Participants had a wide range of circumstances and socio-economic characteristics (Table 2). In contrast to previous studies where older people receiving DPs had been introduced to them via direct payments support schemes or disability groups [38], or at the direct request of family members [49–52]; two-thirds of the sample had only found out about DPs through social or health service sources (Table 1).

**Table 1:** How service users were introduced to direct payments

How aware of DPs	N	Percentage (%)
Social Worker	46	57
Friend	8	10
Publicity (National or local)	5	6
NHS worker (nurse, GP …)	5	6
Not known	4	5
Disability group	3	4
Direct Payments Support Service (DPSS)	3	4
Relation	3	4
Older people’s advisory service	2	3
Domiciliary care agency	1	1
Housing warden	1	1
**TOTAL**	81	100

### Ethical considerations

The research was undertaken before implementation of the Research Governance Framework (2005–2007); its design and methods were reviewed by the corresponding University Research Ethics board, as per guidance at the time. Interviews were conducted face-to-face and older people with cognitive impairment were included (30% of the sample). All but one of these interviews was conducted by proxy with their main representative in the presence of the service user. The person receiving services was addressed, according to their capacity to participate.

Proxies were the unpaid carers managing direct payments as there was usually no other person available with sufficient knowledge of the circumstances to complete the interview. This approach is consistent with other studies [25, 51–53].

For ethical reasons it was stipulated that the main interviewee could not be an unpaid carer remunerated through DP to provide care; in the only case of this a representative from the local Direct Payments Support Services^3^ (DPSS) [54] was called upon. A DPSS representative was also present in two other interviews with service users who lived alone, at their request.

The research was undertaken prior to implementation of the 2005 Mental Capacity Act which extended the scope of DPs to people who lacked capacity to consent and legitimized the practice of authorising a ‘nominated person’ to act on their behalf [55]. Where carers acted for service users unable to express their views, the assumption of responsibility to manage the DP took place under the auspice of lasting power of attorney.

### Measures

All data was obtained during face-to-face semi-structured interviews lasting between 1.5 and 2.5 h in length based on an interview schedule developed for the study (cf. supplementary material 1). The contribution of DPs to outcomes was measured using an adapted version of the Older People’s Utility Scale for Social Care (OPUS [56]), measuring expected outcomes along seven domains: food and nutrition; personal care; safety; social participation and involvement; control over daily living; control over home environment; leisure pursuits/social participation. The last two domains were added to the five-item OPUS; subsequently this tool has been developed to incorporate these extra items (ASCOT [57];). ASCOT is now used in national monitoring of service outcomes [58], and has been subjected to rigorous construct validity testing with older people, including proxies [59, 60].

The interviewer asked to evaluate expected level of need (none, low-level or high-level) in each domain *in the absence of* publicly funded social care (but not excluding freely provided unpaid care) to determine baseline need. As all individuals were receiving a service at the time of interview, evaluation was based either on experiences directly prior to receiving the service or, on experiences of short-term breakdown in care support.

A second need measure for each domain *in the presence of* publicly funded social care input was recorded, related to net outcome of all care inputs (Table 1). The analyses in this study focus on the *difference* between baseline and service impact assessments: hereafter the DP outcome gain (DPOG).

Other data obtained included: reliance on DP support services and/or unpaid carers to manage DPs; how the DP-supported care package was used throughout the week (based on diaries cf. [61]); total care input (including unpaid carer), self-funded care and any support commissioned directly by the council and not part of the DPspackage; activities of daily living (ADL) and instrumental activities of daily living (IADL) scores [62, 63] and dependency level [64] - categorised as low, moderate, moderate-high (2–4 personal activities of daily living (PADLs), high (5 PADLs) or highest dependency on the basis of ADL/IADL item scores and observation during interview.

All measures used in the model are described in the supplementary material (cf. supplementary file 2).

### Analysis

Individual-level analysis of DPOG was conducted using multiple regression analysis. Outcome gain scores had a low mean and were positively skewed; therefore the Poisson log-functional form with a GLM command was used [65].

**Fig. 2:**
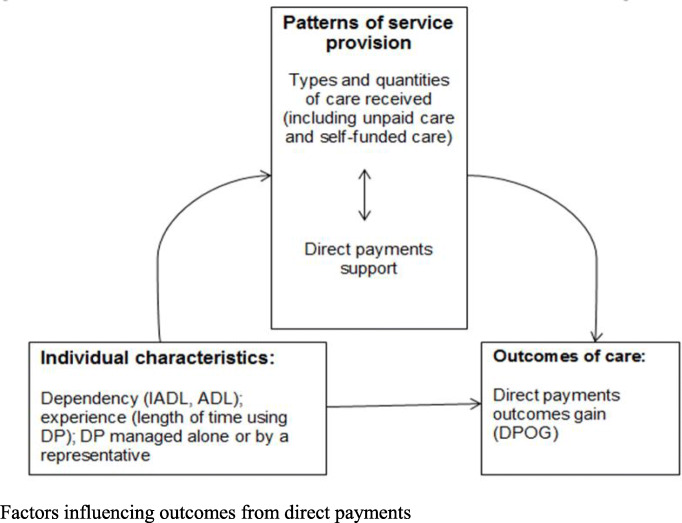
Factors influencing outcomes from direct payments

The model was developed in line with a conceptual framework which hypothesized that outcomes would be influenced by a mixture of individual characteristics (dependency, how DPs where managed) and patterns of service provision (types of care received, direct payments support) (Fig. 2). Given the relatively small sample size, the model was conceived for explanatory purposes [66].

Explanatory variables included individuals’ characteristics, needs (IADL, ADL), dependency and services used. Information on types of support purchased, total care input and proportion contributed to total care input by each support type were included. Total care input represents the weekly sum of hours of: DP support, self-funded care and unpaid care. Hours of care were generally recorded as per the care plan/DP records, but if these differed from the daily diary, the latter took precedence, although the ‘official’ care package amount was recorded separately.

Although data on cognitive impairment was collected (by observation), it was not included as a variable in the model, as it was outside the capacity of the research to include a formal assessment of cognitive impairment, and because of its potential impact on other variables.

A number of variables initially included in the model were later discarded as not statistically significant. These included age > 80; PA turnover, package size (as hours per week and as £ per week), purchased care from a home care agency, percentage of package spent on/ total care input (for all care categories), use of and significance of accountancy service, IADL score and individual scores for the following IADL items: telephone, household tasks shopping, transport. The final set of variables included was a result of a step-wise process, in which attention was paid to avoid collinearity. Risks of overfitting were reduced due to the fact that there were almost no missing data points [67]. Overdispersion was discounted performing the likelihood ratio test of the over-dispersion parameter alpha using a negative binominal distribution.

## Results

### Sample

A third of the sample of 81 people were aged under 71, half 71–85, and 19% over 85 (Table 2); 46% lived alone and 63% were female. Approximately 73% of the sample received unpaid care support to manage their DP to varying degrees, while 43% had their DP fully controlled by an unpaid carer owing to their inability to do so (advanced frailty, limited speech and/or cognitive impairment).

**Table 2:** Sample characteristics

Variable	***n***	Sample all	Moderate dependency	Moderate-high dependency	High dependency	Highest dependency
***n***		***81***	***10***	***13***	***32***	***26***
**(%)**		100	12	16	39	44
***Socio-economic characteristics***						
		**%**	**%**	**%**	**%**	**%**
Age (years)						
< 70	***25***	31	80	24	28	40
70–85	***40***	49	20	12	45	23
85+	***16***	20	–	25	31	44
Gender						
Male	***30***	37	50	38	22	50
Female	***51***	63	50	61	78	50
Lives alone	***38***	48	50	57	53	31
Cognitive impairment^+^	***24***	41	40	15	34	65
Interviewed by proxy	***23***	28	30	23	22	38
Unpaid carer helps to manage DP	***53***	73	50	57	75	88
Ethnicity: BME	***17***	22	30	23	22	19
**Care package values**						
Hourly DP rate **(£)**	***81***	9.46	7.65	11.04	8.06	11.12
Weekly allocation **(hours)**	***81***	20	20	11	19	30
Weekly care package value **(£)**	***81***	189	153	121	153	333
Unpaid care **(hours)**	***70***	33	19	26	30	45
**Care suppliers**						
Unpaid carer	***70***	86	70	85	87	92
Personal assistant(s)	***64***	86	100	82	80	84
Home care agency	***18***	22	–	31	25	23
Privately funded care	***20***	25	–	31	21	35
**Level of met needs**						
Food and nutrition	**79**	93	90	100	92	90
Personal care	**79**	92	90	100	93	85
Safety	***79***	76	90	77	70	65
Social participation	***79***	70	80	62	60	77
Control over daily living	***79***	83	80	84	93	73
Home environment	***79***	85	100	77	83	80
Social and leisure	***79***	65	70	62	66	61
**Other outcomes**						
Feels confident in the event of an emergency	***81***	71	70	85	56	73
Feels more confident in event of an emergency than when using standard services	***48***	95	100	100	85	95
Hospitalized unexpectedly in previous 12 months	***81***	40	30	38	50	42

^+^ Suspected or diagnosed

Most individuals exhibited significant levels of disability: one-third were immobile or chair-bound, two-fifths required assistance with five PADLs and either used a wheelchair or were unable to walk > 2 m (Table 2). Approximately 85% (*n* = 69) of sample members were unable to bath alone, 32% could not use the toilet independently (*n* = 26), and 21% (*n* = 17) and 30% (*n* = 24) were regularly incontinent of faeces and urine, respectively. More than three-fifths were unable to manage finances on their own (*n* = 49), hence particularly likely to require support with DP management. Around 30% of the sample had some degree of cognitive impairment (Table 2); half of which was advanced. These people relied entirely on unpaid care for DP management. Some individuals were in the so-called “grey area” for continuing care funding. However, to receive a DP they had to be solely funded by social care, a situation now altered by availability of personal health budgets (PHBs) [68].

Although the high dependency of sample members reflected the increasing dependency of older people in receipt of state-funded social care, the sample was particularly skewed towards the very dependent. In a 2005 home care sample of 365 people, [69], highest dependency service users comprised 10% of the sample, versus 44% in the current sample. According to social workers interviewed as part of the wider study, this reflected the composition of older DP users at the time, dominated by very complex cases.

Unsurprisingly, DP care packages significantly exceeded ten hours support per week, the Department of Health & Social Care (DHSC) threshold defining intensive community care. Levels of care were particularly intense for the most dependent users, averaging 30 h per week of support (Table 2).

Unpaid care inputs were positively associated with dependency, and varied with nature of relationship between carer and service user: spouses of individuals with high or highest dependency typically provided > 20 h support per week (DHSC threshold for intensive unpaid care) and often > 40 h (Table 2). Spouses (both male and female) represented one third of unpaid carers present (*n* = 27); others were daughters (24%, *n* = 20) and sons (22%, n = 20).

### Outcomes

Net outcomes of all care inputs were generally high, varying by domain (Table 2). Levels of met need were greatest for domains prioritised by state-funded social care, such as food and nutrition and personal care and outcomes were significantly higher than for “supplementary” domains, such as social participation and leisure activities. Needs associated with the home environment (lower-priority domain) were also largely met. Outcomes for the safety domain were especially affected by dependency level, with 28% of the most dependent reporting some unmet need, versus only 10% among the moderately dependent (Table 2).

To put these results into a wider context, the sample outcomes were compared to national outcome data from the Adult Social Care Framework (ASCOF) returns published since 2010/11 when national outcome data was first collected (Table 3). This was a complex task and several factors need to be taken into account in the interpretation of the results:

1. National data for all domains, except one, merge the results of two response options (option one: “no need/ ideal state” and option two: “trivial needs”). This combination provides, “the measure on those individuals achieving the best outcomes, identifying no or limited need” [11, 70], a lower threshold than applied for the DP sample which only reports the percentage of service users who declared that *all* their needs were met (i.e. option one). For simplicity, the terms ‘medium’ and ‘maximum’ threshold are used when comparing the two sets of results (national data versus the DP sample).
2. There is one exception to this rule. National scores for ‘social participation’ are *directly* comparable with the results of the DP sample as both refer solely to responses to option one.
3. From 2016 to 2017 onwards only three domains are covered. This coincides with the introduction of a weighted measure for all domains as a single figure to compare local authority performance. From this date onwards ASCOF returns only detail three domains separately, ‘safety’, ‘social participation’ and ‘control over daily living’ (Table 3).

**Table 3:** Comparison of levels of met needs between DP service users sample and adult social care users according to national data

	Average percentage of service users reporting met needs, by working definition^1^										Average diff. Between sample score and ASCOF scores for all user groups for all time periods^h^
	Sample	Adult Social Care Outcome Framework (ASCOF) results									
	DP Users (2005–2007)	2010–2011 ^(**1)**^	2011–2012 ^**(2)**^	2012–2013 ^**(3)**^	2013–2014 ^(**4)**^	2014–2015 ^(**5)**^	2015–2016	2016–2017	2017–2018	2018–2019	
Food and nutrition	**93**	93	93	93	93	92	92	–	–	–	0.2
Personal care	**92**	93	93	92	93	92	93	–	–	–	−0.7*
Safety	**76**	61	62	63	64	67	67	71 ^a^	71 ^a^	71 ^a^	8.0***
Social participation	**70**	*43*	*43*	*43*	43 ^b^	44 ^c^	45^d^	43^e^	44^f^	43^g^	26.5***
Control over daily living	**83**	73	73	75	74	75	74	75 ^a^	75 ^a^	74 ^a^	9.0***
Home environment	**85**	93	93	93	93	93	94	–	–	–	−8.2***
Leisure/ occupation	**65**	61	63	64	65	66	67	–	–	–	0.7

^1,2,3,4,5,6^ Sources are: 70,71

^a^Sources are: 72, 73

^b,c,d,e,f,g^ Sources are:75,76,77,78,79,80, respectively. National average outcomes for social participation for the years 2010–2013 are estimates

^h^ Results of a paired samples T-test (alpha level 0.05)

*p = < 0.05

***p = < 0.001

Sources: 187, 188

Notes

National average outcomes for the domain of ‘control over daily living’, the domain of ‘safety’ from 2016 onwards and ‘social participation’ report actual figures for over 65’s from 2014 to 2015 onwards. All other figures are adjusted for over 65’s with a 2% reduction of the published national average, to adjust for differences between reported levels of met need between under and over 65’s using values for ‘control over daily living’ from 2015 to 2016 onwards as the reference

There appears to be a slight upward trend in adult social are users reporting some or complete unmet needs from 2014/15 onwards. Data from this point onwards includes service users who fully fund the cost of services themselves. Prior to this time these clients were not included

Starting with the results which are *directly* comparable, levels of met needs for the domain of ‘social participation’ were 26 percentage points greater (95% [CI 24.1–26.2], p. < 0.001) for the DP sample than nationally recorded averages throughout the past decade.

Where the comparison of results is between the ‘maximum threshold’ DP sample responses with the ‘medium threshold’ national results, the comparative performance of DP is understandably compressed. It is particularly striking therefore that met needs among the sample of DP users for the domain of ‘control over daily living’ and ‘safety’ outperformed national average outcome scores *at any time* since data were first recorded by an average 9 and 8 percentage points respectively (95% [CI 8.1–9.9], p. < 0.00195%; [CI 5.8–10.7], p. < 0.001).

For the ‘food and nutrition’ and ‘leisure/occupation’ domains the sample result was roughly equal to the reported ASCOF averages, even though this too compares the maximum needs threshold (DP sample) to the medium needs threshold (ASCOT sample averages).

Finally, the sample averages for the domains of home environment, and personal care, were slightly lower than the ASCOF averages, both of which were statistically significant differences. For the latter the difference was less than one percentage point. Again this compares unequal thresholds (maximum versus medium).

Uniquely for the domain of ‘social participation’ we have some insight into the difference between reported national averages for “no need/ ideal state” and “trivial needs” (maximum/ medium) as results are published by two different sources, one using the medium threshold, and one the high threshold [54, 70–79]. Using this method (which is limited to the years 2013–2014 to 2018–2019), there was a large 31 percentage point difference in the average achievement of only “trivial needs” remaining, versus “no need/ ideal state” for social care recipients at the national level. On this basis it seems fair to conclude that if ‘like-for-like’ national outcomes were available across all domains, the DP sample would almost certainly outperform national average scores across all domains.

Finally, in relation to other measures of quality (Table 2), 83% of the sample with previous experience of standard services felt that services received through DP were *much better*, and 91% felt more confident in the event of an emergency since using DP. For those purchasing care from a home care agency (*n* = 18), 87% felt the agency responded better to their needs as a result of being the direct purchaser. Rates of hospital admission in the preceding 12 months were similar to the *general* population of older people, rather than those with chronic health problems, for whom rates are usually much higher [80].

### Factors contributing to outcomes

#### Service user characteristics

Factors associated with DPOG were examined (Table 4). A strongly significant factor was dependency, consistent with previous findings: those with greatest need derived less benefit from the same amount of service than those with lower needs [81]. Older people living alone reported outcome gains 23% lower than older people living with others (Table 4). Living alone is frequently referred to by social workers as a factor limiting potential benefits of DPs [25]. Living alone and having sufficient ADL difficulties to receive state-funded care can cause social isolation, while older adults living alone are simultaneously more likely to have limited access to unpaid care.

**Table 4:** Factors associated with direct payments outcome gain scores among older people

	Coeff	Prob	95% CI lower limit	95% CI upper limit
Highest Dependency	−0.75	0.00	−0.93	− 0.55
High Dependency	−0.58	0.00	−0.71	− 0.45
Moderate- high Dependency	−0.27	0.00	−0.40	− 0.15
Lives alone	−0.22	0.00	−0.29	− 0.15
Adapted IADL: medication use	0.13	0.00	0.08	0.18
Unpaid carer helps to manage DP	0.13	0.00	0.04	0.21
Chose & received recruitment support service(s)	0.07	0.00	0.05	0.09
Adapted IADL: handling finances	0.08	> 0.01	0.03	0.13
Activities of Daily Living Score	−0.06	0.00	−0.08	− 0.05
Significance of recruitment support (critical)	0.017	0.01	0.004	0.03
Length of time using direct payments	0.003	0.00	0.002	0.0045
Difference between package size and total care input	−0.004	0.00	−0.005	− 0.002
Percentage of total care input composed of self-funded care	0.003	> 0.01	> 0.001	0.005
Percentage of total care input composed of unpaid care	0.006	0.00	0.004	0.008
Percentage of package spent on combination household care/ personal care	0.002	0.00	0.001	0.003
Percentage of package spent on combination household care/ social and leisure care	0.004	0.00	0.002	0.007
Percentage of package spent on therapeutic management	−0.003	< 0.01	− 0.005	−0.001
Constant	4.62	0.00	4.30	4.95

*Observations = 79 Pseudo R*^*2*^ *= 30%*

*GLM model; Link function: Log, Variance function: Poisson*

Alongside dependency level, single IADL items were included. A standard IADL score of 4 and above is a reliable predictor of 1-year incidence of dementia [82]. Scores for each item were adapted so that being autonomous for medication was scored lowest and incapacity for medication scored highest (range 1–5). Individuals with largest adapted IADL scores were more likely to achieve greater outcome gains from DPs. This finding was counter-intuitive: cognitive impairment is a risk factor for package breakdown [83]. This finding probably reflects how individuals lacking these capacities received support by unpaid carers in planning support arrangements, which may therefore indicate the added value associated with ‘managerial care’ performed by unpaid carers [84].

#### Care packages

National statistics show that DPs to older people are less generous than packages to younger adults with physical or learning disabilities, which has raised concerns [26]. Package size was close to significance but exerted little influence relative to other variables and was therefore excluded. However, there was a significant negative association between package size and total care input (Table 4), which may point to a negative impact where there is inequity in social care provision relative to unpaid care input, usually in cases of cognitive impairment and/or extreme dependency. At the time, such individuals were unable to receive health funds as cash payments, a situation now reversed by the 2014 Care Act which permits contribution from NHS continuing care funding to DPs [68].

#### Experience with DPs

Deriving greater outcome gain from DPs was linked to time using the service (Table 4). Using an agency to purchase care was not statistically significant (possibly due to low uptake– only 22% (*n* = 18) of service users purchased care from an agency).

Impacts of care worker characteristics were investigated qualitatively: individuals were asked about continuity, flexibility, reliability, communication, staff attitudes, staff skills and knowledge. Individuals with longest experience using DPs had, for obvious reasons, greatest experience and competence in finding staff. Staff turnover was not relevant to outcome gain (hence excluded from the model).

#### Types of care received

Compared to the characteristics and circumstances of people using DPs, care inputs had less impact on outcome gains, but there were some notable findings. Input of a Direct Payment Support Service (DPSS) was the most influential on outcome gain. Of the two forms of DP support explored (accountancy services and recruitment services), only recruitment services were significantly associated with greater DPOG (Table 4). Receipt of such services was fairly widespread: 69% (*n* = 56) of the sample received ongoing DP support. Of these, 41% (*n* = 23) received both accountancy support and recruitment support, while 36% (*n* = 20) opted for accountancy support only; the remainder relied solely on recruitment support (23% *n* = 13). These services were mainly free at the point of use: only 12% (*n* = 10) of those who used ongoing DPSS support paid towards the cost of the service. Service users were typically referred to the service by the local authority. Referral to DP support was high - in other research it has been found that only one third of DP users ever had contact with a DPSS due to poor referral rates [16].

Of the purchasing choices made, using funds to purchase “therapeutic care” (*n* = 5) was associated with lower outcome gains possibly due to the incidence of cognitive impairment among those purchasing care for this purpose (100%).

DP’s were also applied to purchase “combinations of personal care and home (household) care”. This combination, which was quite frequent (42%,* n* = 34), represented flexible care and contrasted with purchasing care which was solely for home (household) care. The later was not associated with improved outcome gains while the combination purchase was. Purchasing a combination of personal care and home (household) care was linked to hiring a PA: 84% of individuals who received combined personal/ household care hired a PA (*n* = 32); versus only 5% of those that recruited via a home care agency (*n* = 44). A high proportion of service users who recruited a PA (*n* = 64), had received some form of DP support (76%, *n* = 48), with 60% of this group (*n* = 29) receiving recruitment support. Service users who viewed recruitment support as critical also achieved better outcomes (Table 4). Surprisingly, 50% of those who did not recruit a PA (*n* = 12), also used recruitment support. In these cases, DPSS acted as brokers for individuals purchasing care from home care agencies.

These findings help to better understand the role of DP support as an ‘intermediate output’ in the production of DPOGs [85]. Previous research has noted that ‘third-party organisations’ support improves outcomes for individuals with and without unpaid care [30, 86]. Backed by qualitative research it has been widely accepted that DP support is critical to take-up of DPs [87], that absence of payroll support can put people off using DPs [38] and that DP support can ease the burden felt by unpaid carers [36, 88] but the lack of a clear association between the role of support services and particularly support for recruiting care (as demonstrated in this study), has weakened the priority given to DP support.

Last but not least, individuals receiving privately-funded care (25% of sample) had better outcomes. Overall self-funded care was marginal to total care input received but exceeded 25% of total care input in the following subgroups: highly-dependent users who either lived alone (regardless of whether or not they had some form of unpaid care); people who did not live alone but self-managed their DP, and people who received no unpaid care. In essence, self-funded care offered a substitute to unpaid care. Despite the term, ‘self-funded care’ was predominantly publicly funded, albeit indirectly by service users employing their Attendance Allowance to purchase extra care. This is a social security benefit widely available to older people requiring regular care or supervision. Around 1.24 million older people in England receive Attendance Allowance, compared to around 411,000 who receive some form of local authority adult social care support [89]. Attendance Allowance (AA) was used equally among those with and without unpaid care, often prompted by advice from support workers.

## Discussion

### Importance of unpaid care

A major thread running through the results is the positive influence of unpaid care on DPOG. This corroborates the views of social workers [25, 90], but the analyses presented identify how and why unpaid care is so influential.

#### Unpaid care as a function of total care

A positive association between DPOG and receiving a higher fraction of total care input from unpaid care may *seem* unsurprising but actually the situation is complex. Unpaid care as a fraction of total care can limit potential outcome gain from state-funded care, as need in the absence of a service may be reduced.

There is a longstanding debate as to whether unpaid care complements or substitutes for formal care [91]. Cash payments may decrease unpaid caregiving if families have greater license to organize care to suit their priorities [60]. The results challenge these concerns and strongly suggest that in the context of DPs in England^4^ unpaid care not only complements formal care, but promotes its efficacy.

The Care Act (2014) expressly aims to reduce carer burden; a question then arises about the appropriateness of a service indirectly promoting unpaid care. Evidence for recent increases in intensive caregiving among over 65 s is available [92]. Within the sample, unpaid care contributed on average 42% of the total care input when available [44].

At the individual level the benefit of DPs depends upon whether or not DPs offer what carers are lacking, such as the ability to coordinate care to fit in with their other responsibilities [93]. There is largely consensus that DPs to the person being cared for can assist unpaid carers in gaining more control over their time and daily lives and improve their quality of life [38, 94, 95]. However, managing a DP should not be imposed due to a lack of alternatives [95]; crucially the pre-existing relationship between carer and recipient should be taken into account when considering the option of DPs.

#### Dependency on an unpaid carer to manage the DP

Research on situations where the unpaid carer manages a DP making proxy decisions, has mainly focused on how this role comes about [49, 50, 52], and whether practitioners are confident at assessing when this arrangement is in the person’s best interest [49, 50]. These results are the first to offer quantitative evidence linking DP management by an unpaid carer with better outcomes for the cared-for person. This discounts concerns that care may become ‘carer-centered’ [95] and validates previous suggestions that there is often considerable overlap between the needs and goals of the cared-for and the carer [96, 97].

It has been speculated that the responsibility for managing care may overburden already stretched caregivers [98] but equally involvement in coordinating care (facilitated by availability of DPs) may increase the ‘process utility’ of caregiving [99]. Attributes such as ‘control over the caring’, ‘fulfillment’ [100], and “a sense of control and mastery” [101], are known to promote carer wellbeing. It has also been found that unpaid carers can simultaneously perceive both moderate burden and great satisfaction [102]. None of these aspects have yet been adequately explored in relation to DP management.

### Influence of the type of inputs received

The findings also highlight the influence of the type of inputs received (Fig. 2); throwing light on the inputs characteristic of more flexible care, and on the positive impact of direct payments support (DPS).

#### Flexible care arrangements

Individuals purchasing flexible care, (i.e. marginally deviating from standard home care), achieved greater gains. This was most prevalent among service users who used a PA which is unsurprising, as employing a PA has long been considered the best route to greater autonomy [103] but this is the first study to demonstrate quantitatively that flexibility can improve outcomes.

Research has reported a decrease in the use of PAs [46, 104] and increasingly narrow interpretations of what would be “appropriate use of funds” [25, 105]. The majority of those that recruited a PA had done so with some form of support from a DPSS.

#### Support structures

Recruitment support provided by DPPS significantly altered outcomes. Those that used recruitment support had better outcomes. Furthermore, those who considered recruitment support as critical to their success – had even better outcomes. These findings are important given the way that recruitment support has been reconfigured in many areas with reductions on the ‘associated expenditure’ of DP support by decommissioning and a shift towards online platforms. In an increasing number of councils service users are expected to make a choice about whether they should dedicate a portion of their DP to pay for a potentially beneficial support service, without knowing in advance what that might mean for them [44]. This scenario contrasts with the access to DP support that many of the service users in this sample had: free at the point of use, 1:1 and allowing service users to explore options regardless of the means by which they eventually recruited care. The results suggest that these changes are likely implicated in the increasing failure of DPs to achieve better outcomes than standard services.

### Financing DPs: sufficient?

For some time researchers have argued that DPs to older people may be of insufficient value to achieve optimal outcomes [26]. The results challenge this argument in that package size was not statistically linked to DPOG but this only has weight if the intensity of care packages for the sample were consistent with practices at the time, and comparable to recent levels of per capita expenditure. This is difficult to ascertain given wide variations in expenditure between councils, both then and now, but some observations can be made.

In terms of the overall sample, we see that average per capita expenditure was £189 (Table 1) in 2006. Currently, the average per capita expenditure on DP among the over 65 s is £266 [19, 20]. Taking into account current hourly home care costs, (£16.04, 157b) this equates to roughly 16.5 h of state funded social care per week in 2018–2019, versus 17 h a week for the sample based on average unit home care costs in 2006 (£11, 158). The average weekly DP value for those of highest dependency (44% of the sample) was £333. This averages to 30 h of state-funded social care per week, consistent with the intensity of package size at the time for those levels of dependency [106].

While this parity underlines the relevance of the results to current practice, it does not rule out concern regarding today’s funding levels. Average DP package values for older people have generally risen over the past four years, with a median increase of 19% but with large variance (±65%) [20, 107–109] within which there is ground for concern. It is debatable whether the rise in funding is sufficient to fund sustainable care from the home care sector [110].

Focusing on what can be asserted from the findings, it is evident that the sufficiency of DP packages can only really be understood in relation to other factors. Whilst variations in outcome gain were not significantly associated directly to DP package values, outcome gains were reduced where there was a larger *discrepancy* between the total care input (which could include unpaid care and/or self-funded care), and the funded package. Larger discrepancies were observed where (a) individual allocations of DPs had been capped (b) service users were physically able but cognitively impaired and received DP amounts that were minimal relative to their needs.

These effects were not a direct result of shifting a greater burden onto unpaid care, or of a greater responsibility to self-fund. In fact, service users’ for whom either unpaid care or self-funded care represented a higher fraction of the total care input had greater DP-related outcome gains. It appears that DP’s were less effective simply for being out of line with individuals’ circumstances, as a consequence of legal constraints (extra funding from health was still not legally permitted, hence the cap) or lack of fit with existing resource allocation practices.

The results suggest therefore that DP package values *do* influence outcomes, but the effect is weak against other factors, provided funding is set at an *appropriate* level (for which the current sample might give a benchmark value, if properly uprated). Certainly, large variations in per capita expenditure would result in major (negative) deviations from benchmark values and in packages likely to be misaligned with individuals’ circumstances. There also appears to be significant potential for optimizing DPOG in mobilizing NHS contributions where applicable.

### Living alone and “resource-poor”

As expected, living alone was associated with worse DPOG. Social networks have also been associated with better outcomes from PBs [31]. The different mechanisms by which unpaid care influenced outcome gains show that those without a carer were resource-poor in various ways. Unpaid care was influential both as a function of total care received as a commodity, and from a ‘capabilities’ perspective [111] with unpaid carers acting as agents.

There were also other inequalities between service users. People receiving care from other sources gained more from the DP funded part of their care, than those that did not. Care from ‘other sources’ included self-funded care, financed mainly by Attendance Allowance, often prompted by advice from support workers.

DPs had their limitations in outcomes for certain domains (Table 1). Like recent reports of unmet needs in self-directed HCBS programs [112], qualitative interview data suggested other relevant social issues – such as inability to use transport, lack of interest in attending organized groups or lack of acceptable meeting places – coupled frequently with a general demotivation, related to the loss of siblings and peers. Also, there were significant needs in the domain of home environment, from basic decoration to adaptations that social care funds would not meet. It is unlikely that these needs may have been met simply by access to more generous care packages, but might have been eased by other social care interventions [113, 114]. Still there are now increased opportunities for using DPs as vehicles for tackling these issues: home equipment and adaptations now lie within the realm of DP.

#### Limitations

The data used for the analysis was cross-sectional and some caution needs to be taken in its interpretation in the absence of longitudinal data. The analysis also combines proxy with non-proxy responses. While this is not unusual it has some limitations. Separating the two sets of responses would be a complex issue, requiring a much larger sample. Proxy responses were for obvious reasons biased towards the most dependent thus making it difficult to control for differences. The potential influence of unpaid carers on outcomes scoring was also not just limited to proxies. Just under a third or the interviews were conducted by proxy - but the majority of the interviewees received some degree of support from an unpaid carer to manage their DP (73%) and in most of these cases their unpaid carer was also present in the interview.

The use of proxies was paramount to achieving a sample better matched to the levels of dependency currently supported by publicly-funded social care than survey data. It is a widely used method for collecting data, “preferable to the systematic exclusion of individuals who are unable to self-report based primarily on the principles of equity and inclusion, as well as the potential methodological issues associated with missing data and bias” [2, 59].

The strong positive impact on outcomes associated with the presence of an unpaid carer who helped to manage DPs clearly prompts reflection on the potential of *positive* bias linked to proxy responses but, “the majority of studies that directly compare self-report and proxy-report have found an underestimation of quality of life by proxy respondents compared to patient self-report” [12, 59]. It therefore seems unlikely that proxies overestimated outcomes. Proxy evaluation of DP outcomes was also strengthened by independent observation [25].

## Conclusion

The work presented has explored how outcomes are influenced by the types and quantities of care purchased; external support to manage DPs (from DPSS and from unpaid carers), as well as individual characteristics. Unlike previous survey data which excludes proxy responses [23, 31], service users in the sample were skewed towards the most highly dependent. The sample therefore better reflects the profile of older people currently receiving publicly-funded social care. The payments received by the sample were also in line with current norms. Outcomes of DPs for the sample were compared with national average outcome scores across a nine year period (since reporting began), and tested for statistically significant differences. This was a complex task owing to different methods of coding met needs; for most domains the DP sample compares “all needs met” (maximum threshold) with national results which combine “no needs” and “only trivial needs” (medium threshold). Despite these threshold differences there were strong statistically significant differences in the extent to which the DP sample felt safe and in control of their lives and achieved as much social participation as they wanted. If data was available to compare the two at the same threshold, the DP sample results would have likely outperformed national average outcome scores for all domains.

The findings are historical – based on interviews conducted between 2005 and 2007. The revisiting of this data is justified on two counts. The data offers more detail than previous studies – but also the very fact that it predated the main wave of personalisation is an advantage. Personalisation has radically altered the context in which DP are used by older people and reports of decreasing success of DPs to older people coincide with the period associated both with the implementation of personalisation and radical austerity [42]. The richness of the data provides the opportunity to explore possible reasons for this.

Two particular aspects stand out: flexible care and support structures. Inputs characteristic of more flexible care were associated with achieving greater outcome gain from receiving DPs. (Conversely using DPs to purchase “mainstream” support would not be expected to do so.) Also individualized support provided by a dedicated DPSS increased the benefit associated with DPs. This is where the major changes in DP implementation and support that have occurred in recent years [44], may be relevant. Such changes are likely to have impacted upon the inputs received and the characteristics of people receiving DPs. This is likely to have occurred directly (i.e. excessive limitations on use of funds causing reduced flexibility; reduced access to face-to-face recruitment support akin to the support received by the sample in this study), as well as indirectly (such as by creating environments where employing a PA is more difficult, or influencing who gets DPs).

A major concern surrounding the uptake of DPs in the wave of personalisation and austerity is that current pressures and incentive structures promote the ‘easiest’ rather than the best route of care. This, for an increasing number of councils, equates to DPs being supplied as the ‘default’ option. Due to the pressures on social work teams this often precludes access to DPs in so called ‘complex-cases’. These include: services users requiring indirect payments (managed by a nominated person), particularly people with dementia, individuals requiring health funding and people for whom including funds to purchase home equipment and adaptations may be beneficial. The results of the DP sample support the promotion of DPs for complex-cases but highlight the need to pay attention to the discrepancy between total care input (which could include unpaid care and/or self-funded care) and DP-funded support. This information is not routinely collected but could be required as a means of monitoring – particularly given concerns that DPs offer a convenient route to councils to further shift caregiving costs to unpaid carers.

Providing adequately sized care packages for complex cases requires an increased role for funding from NHS continuing care, made legal as part of the Care Act 2014 [68]. Implementation of Personal Health Budgets (where direct payments combine social and health care funding) has been marred by the unwillingness of NHS commissioning groups to release funds to councils with social services responsibilities. As a result, service users at the high end of the need spectrum, as represented in the sample, are less likely to access DPs.

Consistent with earlier qualitative studies, the work found positive impacts of unpaid care on older DP recipients but this is the first study that quantifies this, and demonstrates separate effects for unpaid care as a function of the total care received, and unpaid care as managerial care. The findings provide an incentive to recognise the often overlooked impact of unpaid carers on the outcomes of DPs [23]. Assuming that “if the service user [is] unable to manage a DP, then the carer [will] be asked to manage it for them”, [52] really is the wrong approach. We know this can negatively affect carer wellbeing [115]. This study also shows that just having an unpaid carer is not necessarily sufficient: it is the time and effort that they invest in caring that is significant. This insight helps with the dilemma regarding overreliance on unpaid carers. Unpaid carers’ commitment and capability can (and should) be readily observed at the outset.

Finally, this work demonstrates for the first time that the freedom to combine care package allocations with self-funding is associated with achieving better outcomes. DPs remain the only mechanism by which service users and families can choose to add to their funded package, but in the past this has provoked heated debates about the risk of a two-tiered service [116]. In this study, self-funded care was a small but pivotal factor in optimizing outcomes. It was also predominantly funded by the social security benefit Attendance Allowance. This benefit remains surrounded by controversy amidst discussions on the future funding of social care [117]. Its proponents point to its wide coverage; ability to compensate for unmet need among people who remain ineligible for social care funding and the value of it being centrally administered at set rates, thus offering some independence from the highly variable practices of local councils.

The work presented provides an urgent reminder that it is not access to DPs per se that improves outcomes but *DPs with support* to identify and realise the potential they offer. It is said that personalisation has not worked for older people [118]. Others argue that suggesting that personal budgets are unsuitable for older people is, in itself, a form of ageism [33]. This work offers insights into the tools at councils’ disposal to improve the potential of DPs, as well as lessons for other countries implementing consumer-directed care.

## Supplementary Information

**Additional file 1.**

**Additional file 2.**

## Data Availability

The datasets used and/or analysed during the current study are available from the corresponding author on reasonable request.

## Abbreviations

PB: Personal budget
DP: Direct payment
MB: Managed budget
DPSS: Direct payments support scheme
DPOG: Direct payment outcome gain
DH: Department of Health

## Footnotes

1 Home care (also termed ‘domiciliary care’) is care provided at home. Where home care is state-funded, it is arranged through the local authority and usually provided by private home care agencies. These agencies recruit and train individuals to provide care per the service users’ support (or ‘care’) plan according to eligible assessed needs. This may include support with personal care, such as washing or dressing; cooking or preparing meals and/ or housekeeping or domestic work. Priority is given to personal care, nutrition and safety needs.

2 The pattern of local government in England is complex, with the distribution of functions varying according to the local arrangements. ‘Council’ refers to a council with social services responsibilities which include: London boroughs, Unitary authorities, Shire authorities and Metropolitan councils.

3 Direct payment support schemes provide support to people receiving direct payments. Services available may include support with: devising a support plan; budgeting; accountancy (and payroll, if hiring a personal assistant), recruitment and employee responsibilities. DPSS may also offer information on local homecare providers for service users who wish to purchase from a homecare agency, rather than recruit a personal assistant.

4 This context includes rigorous limitations on funding relative to need and consideration of available unpaid care prior to allocation of funds.
