# Correction to: Influences of service characteristics and older people’s attributes on outcomes from direct payments

**DOI:** 10.1186/s12877-021-02167-0

**Published:** 2021-04-16

**Authors:** Vanessa Davey

**Affiliations:** 1 Research Fellow, Re-FIT Research Group, Parc Sanitari Pere Virgili & Vall d’Hebrón Institute of Research (VHIR), Barcelona, Spain; 2 Formerly at Personal Social Services Research Unit (PSSRU), London School of Economics & Political Science (LSE), London, UK

**Correction to: BMC Geriatr 21, 1 (2021)**

**https://doi.org/10.1186/s12877-020-01943-8**

Following publication of the original article [1], the author notified about the errors in article text body. The correct sentences should be as follows:

- Older people living alone reported outcome gains 22% lower than older people living with others (Table 4).
- Taking into account current hourly home care costs, (£16.04, 20) this equates to roughly 16.5 h of state funded social care per week in 2018–2019, versus 17 h a week for the sample based on average unit home care costs in 2006 (£11, 40).
