# Supplementary material for: Influences of service characteristics and older people’s attributes on outcomes from direct payments
Source: BMC Geriatr. 2021 Jan 2;21:1. doi: 10.1186/s12877-020-01943-8 (PMC7777219; doi:10.1186/s12877-020-01943-8)
Supplement: Supplementary file 1 — Additional file 1. [file 12877_2020_1943_MOESM1_ESM.doc]

**DIRECT PAYMENTS TO OLDER PEOPLE STUDY**

QUESTIONNAIRE FOR DIRECT PAYMENT USERS

**SECTION 0 –** *TO BE COMPLETED BY INTERVIEWER*

A) Site **[Site]**

⁪ [Site 1] **[1]** 

⁪ [Site 2] **[2]** 

⁪ [Site 3] **[3]** 

⁪ [Site 4] **[4]** 

⁪[Site 5] **[5]** 

⁪[Site 6] **[6]** 

[Site 7] **[7]**  

[Site 8] **[8]** 

⁪[Site 9] **[9]** 

[Site 10] **[10]** 

B) Case per site

**⁪** Interview 1 = 1; Interview 2 = 2… 

C) Does the direct payment recipient has observed cognitive impairment? **[cogimp]**

[COMPLETE AT END]

Yes ⁪ **[1]** 

No ⁪ **[2]** 

C) Interview conducted by proxy? **[proxy]**

Yes ⁪ **[1]** 

No ⁪ **[2]** 

With whom and why?

______________________________________________________________________________________________________________________________________________________

___________________________________________________________________________

**SECTION 1 –** DEMOGRAPHIC DATA

1 How old are you? **[age]**

65-70 ⁪ **[1]** 

71-75 ⁪ **[2]** 

76-80 ⁪ **[3]** 

81-85 ⁪ **[4]** 

86-90 ⁪ **[5]** 

91-95 ⁪ **[6]** 

95+ ⁪ **[7]** 

1.2a Do you live alone? **[livesalone]**

Yes ⁪ **[1]** 

No ⁪ **[2]** 

b) If not, who do you live with? **[liveswith]**

Spouse ⁪ **[1]** 

Daughter or son ⁪ **[2]** 

Grandchild ⁪ **[3]** 

Other relative ⁪ **[4]** 

Friend ⁪ **[5]** 

1.3 To which of these groups do you belong?

White (British, Irish or other white background) ⁪ **[1]** 

Mixed (White and Black Caribbean, White and Black African, ⁪ **[2]** 

White and Asian, any other mixed background)

Asian or Asian British (Indian, Pakistani, ⁪ **[3]** 

Bangladeshi, any other Asian background)

Black or Black British (Caribbean, African or any ⁪ **[4]** 

Black background)

Chinese ⁪ **[5]** 

Any other ethnic group ⁪ **[6]** 

1.4 What education have you received during your life?

GCSE Equivalent (Left at 16 years of age) ⁪ **[1]** 

A-Level Equivalent (Left at 18 years of age) ⁪ **[2]** 

Technical College ⁪ **[3]** 

Degree ⁪ **[4]** 

Higher degree ⁪ **[5]** 

Not applicable ⁪ **[-3]** 

1.5 What has been your occupation during your life?

_______________________________________________________________________________________________________________________________________________________________________________________________________________

**SECTION 2** – ACCESS TO DIRECT PAYMENTS

2.1 How long have you been using Direct Payments?

___________________________________________________________________________

2.2 How did you become aware of direct payments? **[hwawr]**

Through a Social Worker ⁪ **[1]** 

Through disability group ⁪ **[2]** 

Through direct payments support group ⁪ **[3]** 

Through a friend ⁪ **[4]** 

Other (please indicate) ⁪ **[5]** 

______________________________________________

2.3a) Were you previously receiving money from the Independent Living Fund to purchase care? **[hadilf]**

Yes ⁪**[1]** 

No ⁪**[2]** 

Don’t know ⁪**[-2]** 

Missing ⁪**[-1]** 

Not applicable **[-3]** 

b) If so, do you still receive money from the Independent Living Fund as well as money from social services (as a direct payment)? [The Independent Living Fund is available to disabled people under the age of 66 who receive more than £200 per week of services or direct payment]? **[stillilf]**

Yes ⁪**[1]** 

No ⁪**[2]** 

Don’t know ⁪**[-2]** 

Missing ⁪**[-1]** 

Not applicable **[-3]** 

c) If yes, how much do you receive from the ILF? **[ILF]**

Per week ⁪

Don’t know ⁪**[-2]** 

Not applicable **[-3]** 

d) If no, did you experience any delays in reassessment and review when you were no longer

eligible for the ILF? **[reasdly]**

Yes ⁪**[1]** 

No ⁪**[2]** 

Don’t know ⁪**[-2]** 

Missing ⁪**[-1]** 

Not applicable **[-3]** 

2.4a) Did you receive social care and support of the traditional type before participating in direct payments? **[tradssbf]**

Yes ⁪**[1]** 

No ⁪**[2]** 

Don’t know ⁪**[-2]** 

Missing ⁪**[-1]** 

b) If yes, how does using direct payments compare to receiving standard services? **[tradssbf]**

Much better ⁪ **[1]** 

A little better ⁪ **[2]** 

Roughly the same ⁪ **[3]** 

A little worse ⁪ **[4]** 

A lot worse ⁪ **[5]** 

Don’t know ⁪ **[-2]** 

Missing ⁪ **[-1]** 

Not applicable **[-3]** 

Any comments?

____________________________________________________________________________________________________________________________________________________________________________________________________________________________________________________________________________________

c) If yes, which services did you use? **[dpvsss]**

Day care ⁪ **[1]** 

In-house domiciliary care ⁪ **[2]** 

Private domiciliary care agency ⁪ **[3]** 

Residential care ⁪ **[4]** 

Nursing home care ⁪ **[5]** 

Other (please detail) ⁪ **[6]** 

Don’t know ⁪ **[-2]** 

Missing ⁪ **[-1]** 

Not applicable **[-3]** 

d) If you felt that receiving standard services was worse than receiving care through direct payments, could you give an indication of the main problems with standard services?

**[Please rank the following in order of importance. 1 = most important, 7 = least important, -3 = Not applicable, -2 = Don’t know]**

**[USE PROMPT CARD]**

Rank

Carers late or not turning up ⁪ **[crslte]** 

Too many different carers ⁪ **[toomny]** 

Carers’ attitudes ⁪ **[crsattit]** 

Poor quality of care ⁪ **[prqu]** 

Lack of flexibility in support and care provided (i.e. timing, chores) ⁪ **[lkflex]** 

Lack of choice (i.e. provider) ⁪ **[lkchi]** 

Poor level of control ⁪ **[lkcont]** 

Lack of time (being rushed) ⁪ **[lktime]** 

Not applicable **[-3]** 

Anything else?

_________________________________________________________________________________________________________________________________________________________________________________________________________________________________

2.5 Why did you choose direct payments?

_________________________________________________________________________________________________________________________________________________________________________________________________________________________________

2.6 Were you automatically offered the option of using direct payments by your social worker, or did you request them? **[autoffdp]**

Yes, automatically offered direct payments. ⁪ **[1]** 

No, had to request them. ⁪ **[2]** 

Don’t know ⁪**[-2]** 

2.7 How did you find the response of your social worker to your request for direct payments? **[respsw]**

Positive ⁪ **[1]** 

Neutral ⁪ **[2]** 

Negative ⁪ **[3]** 

Don’t know ⁪**[-2]** 

2.8 How satisfied were you with the initial information on direct payments given to you?

**[satinf]**

Very satisfied ⁪ **[1]** 

Satisfied ⁪ **[2]** 

Neutral ⁪ **[3]** 

Unsatisfied ⁪ **[4]** 

V. Unsatisfied ⁪ **[5]** 

Don’t know ⁪**[-2]** 

**SECTION 3** – SUPPORT AND ADVICE

*In this section, I would like to find out which organisations and/ or individuals have offered you support and advice with direct payments. I would like to know how satisfied you are with the support and advice. In the following section I will ask more specific questions about the services that these organisations/ individuals may have offered you and the services you may have chosen to help you manage direct payments.*

3.1 Have any of the following from the Local Authority provided you with support and advice in using Direct Payments? **[advss]**

**[Yes = 2; No = 2; -2 = Don’t know]**

A Social Worker ⁪**[laspsw]** 

An Independent Living Advisor/ Direct Payments Coordinator **[laspdpa]** 

A Payroll Advisor **[lasppa]** 

An Audit Officer **[lapsao]** 

A Finance Officer **[laspfo]** 

A Commissioning Officer **[lapsco]** 

3.2 How satisfied are you with the support given to you by social services? **[satadvss]**

Very satisfied ⁪ **[1]** 

Satisfied ⁪ **[2]** 

Neutral ⁪ **[3]** 

Unsatisfied ⁪ **[4]** 

V. Unsatisfied ⁪ **[5]** 

Don’t know ⁪ **[-2]** 

Missing ⁪ **[-1]** 

3.3 Who from the local authority do you turn to most for support and for what reasons?

_______________________________________________________________________________________________________________________________________________________________________________________________________________

3.4 What difference does their support make to your experience of direct payments? **[sigadvss]**

Could not mange without them ⁪ **[1]** 

Could manage without them but would feel less supported ⁪ **[2]** 

Could manage without them ⁪ **[3]** 

3.5 Do you feel that the level of support you receive from social services is sufficient to prevent you from receiving unsatisfactory standards of care from the care workers you employ? **[supprv]**

Yes ⁪**[1]** 

No ⁪**[2]** 

Don’t know ⁪**[-2]** 

Missing **[-1]** 

a) If yes, what aspects of the support you receive from the local authority are the most important in preventing you from receiving unsatisfactory standards of care?

_________________________________________________________________________________________________________________________________________________________________________________________________________________________________

b) If not, why not?

_________________________________________________________________________________________________________________________________________________________________________________________________________________________________

3.6 Do you feel that generally the function of social services support is to prevent you from receiving unsatisfactory levels of care, or is it to support you in other ways? **[rolssprv]**

To prevent receipt of unsatisfactory care ⁪ **[1]** 

To support in other ways ⁪ **[2]** 

Both ⁪ **[3]** 

Neither ⁪ **[4]** 

Don’t know ⁪ **[-2]** 

Missing ⁪ **[-1]** 

Any comments?

_______________________________________________________________________________________________________________________________________________________________________________________________________________________________________________________________________________________________________________________________________________________________________________________

3.7 Do you feel that the support given social services is generally appropriate or sufficient? **[supssapr]**

Yes ⁪**[1]** 

No ⁪**[2]** 

Don’t know ⁪**[-2]** 

Missing **[-1]** 

3.8 Have you ever been consulted about the type of support that you think should be delivered locally? **[consss]**

Yes ⁪**[1]** 

No ⁪**[2]** 

Don’t know ⁪**[-2]** 

Missing **[-1]** 

3.9 Have you ever been offered or received support and advice with your direct payments from a Local Independent Living Centre or other similar community or voluntary agency? **[recspilc]**

Yes ⁪**[1]** 

No ⁪**[2]** 

Don’t know ⁪**[-2]** 

Missing **[-1]** 

3.10 Have you had contact with a Direct Payments Support Worker via your Local Independent Living Centre? **[recspdps]**

Yes ⁪**[1]** 

No ⁪**[2]** 

Don’t know ⁪**[-2]** 

3.11 How satisfied are you with the support given to you by your Direct Payments Support Worker? **[satdpsw]**

Very satisfied ⁪ **[1]** 

Satisfied ⁪ **[2]** 

Neutral ⁪ **[3]** 

Unsatisfied ⁪ **[4]** 

V. Unsatisfied ⁪ **[5]** 

Don’t know ⁪ **[-2]** 

Not applicable **[-3]** 

3.12 What difference does the support of your Direct Payments Support Worker make to your experience of direct payments? **[sigdpsw]**

Could not mange without them ⁪ **[1]** 

Could manage without them but would feel less supported ⁪ **[2]** 

Could manage without them ⁪ **[3]** 

3.13 How satisfied are you with the support given to your Local Independent Living Centre? **[satilc]**

Very satisfied ⁪ **[1]** 

Satisfied ⁪ **[2]** 

Neutral ⁪ **[3]** 

Unsatisfied ⁪ **[4]** 

V. Unsatisfied ⁪ **[5]** 

Don’t know ⁪ **[-2]** 

Not applicable **[-3]** 

3.14 Have you ever been offered or received support and advice by a peer support group (such as a Personal Assistance User Group). **[ofsppsg]**

Yes ⁪**[1]** 

No ⁪**[2]** 

Don’t know ⁪**[-2]** 

3.15 If so, how satisfied are you with the support given to you by your peer support groups? **[satpsg]**

Very satisfied ⁪ **[1]** 

Satisfied ⁪ **[2]** 

Neutral ⁪ **[3]** 

Unsatisfied ⁪ **[4]** 

V. Unsatisfied ⁪ **[5]** 

Don’t know ⁪ **[-2]** 

Not applicable **[-3]** 

3.16 Have you ever been offered or received support and advice with your direct payments from an Inland Revenue Business Support Team? **[ofspirbs]**

Yes ⁪**[1]** 

No ⁪**[2]** 

Don’t know ⁪**[-2]** 

3.17 If so, how satisfied are you with the support given to you by the Inland Revenue Business Support Team? **[satirbs]**

Very satisfied ⁪ **[1]** 

Satisfied ⁪ **[2]** 

Neutral ⁪ **[3]** 

Unsatisfied ⁪ **[4]** 

V. Unsatisfied ⁪ **[5]** 

Don’t know ⁪ **[-2]** 

Not applicable **[-3]** 

3.18 Out of the organisations/ individuals listed, whom do you turn to most for support and for what reasons?

_______________________________________________________________________________________________________________________________________________________________________________________________________________

**SECTION 4** – ADVOCACY, BROKERAGE AND EMPLOYEE MANAGEMENT SERVICES

*In this next section, I would like to find out what services have been offered to you by the organisations and/ or individuals you were in contact with and which services you choose:*

4.1a) Were you offered an **Accountancy Service**? **[offaccs]**

(Such a service may set up bank accounts, issue cheques, deal with tax and National Insurance and fill in monitoring forms)

Yes ⁪ **[1]** 

Not offered ⁪ **[2]** 

Not available ⁪ **[3]** 

Don’t know ⁪ **[-2]** 

b) Did you choose to receive services from an **Accountancy Service**? **[chaccs]**

Yes ⁪**[1]** 

No ⁪**[2]** 

Don’t know ⁪**[-2]** 

Not applicable **[-3]** 

c) If not, why not?

_________________________________________________________________________________________________________________________________________________________________________________________________________________________________________________________________________________________________________________________________________________________

d) *Which services were you offered?* **[Yes = 1; No =2]**

# Services offered

Help setting up a bank account ⁪ **[offbkacc]** 

Issued Cheques ⁪ **[ofisucq]**

Assistance with tax ⁪ **[ofaswtx]**

Assistance with National Insurance ⁪ **[ofasni]** 

Assistance with Payroll ⁪ **[ofaspyrl]** 

Training in budgeting ⁪ **[oftrnbgt]** 

Completing monitoring forms ⁪ **[ofmntr]** 

Help organising employers’ liability ⁪ **[ofinsur]** 

insurance

Not applicable **[-3]** 

e) *Which services did you choose?***[Yes = 1; No =2]**

# Services chosen

Help setting up a bank account ⁪ **[chbkac]** 

Issued Cheques ⁪ **[chiscq]** 

Assistance with tax ⁪ **[chawtx]** 

Assistance with National Insurance ⁪ **[chain]** 

Assistance with Payroll ⁪ **[chaspyrl]** 

Training in budgeting ⁪ **[chtrnbgt]** 

Completing monitoring forms ⁪ **[chmntr]** 

Help organising employers’ liability ⁪ **[chinsur]** 

insurance

Not applicable **[-3]** 

f) If you choose to receive help from the **accounting service**, what difference does this service make to your experience of direct payments? **[sigaccs]**

Could not mange without them ⁪ **[1]** 

Could manage without them but would feel less supported ⁪ **[2]** 

Could manage without them ⁪ **[3]** 

Not applicable **[-3]** 

4.2 a) Were you offered **Recruitment Support**? **[ofrcs]**

(Advocacy services may provide lists of personal assistants, lists of agencies, financial advice, assistance with compiling job descriptions, assistance with training, assistance with recruiting or general advice and support).

Yes ⁪ **[1]** 

Not offered ⁪ **[2]** 

Not available ⁪ **[3]** 

Don’t know ⁪ **[-2]** 

b) Did you choose to receive services from the **recruitment support service**? **[chrcs]**

Yes ⁪**[1]** 

No ⁪**[2]** 

Don’t know ⁪**[-2]** 

Not applicable **[-3]** 

c) If not, why not?

_________________________________________________________________________________________________________________________________________________________________________________________________________________________________________________________________________________________________________________________________________________________

# d) *Which services were you offered?*

# Services offered

Lists of personal assistants ⁪**[oflpas]** 

Lists of local agencies ⁪**[ofllag]** 

Financial advice ⁪**[offina]** 

Assistance compiling job descriptions ⁪**[ofjbdcr]** 

Assistance compiling contracts ⁪**[ofconts]** 

Assistance with training ⁪ **[oftrn]** 

Assistance with recruiting ⁪**[ofrecrt]** 

Assistance with interviews ⁪**[ofintr]** 

Bank of emergency staff ⁪**[ofemrg]** 

Any other back-up service ⁪**[ofotbu]** 

General advice and support ⁪**[ofgen]** 

Not applicable **[-3]** 

## e) *Which services did you choose?*

## Services chosen

Lists of personal assistants ⁪**[chpas]** 

Lists of local agencies ⁪**[chllag]** 

Financial advice ⁪**[chfina]** 

Assistance compiling job descriptions ⁪**[chjbdcr]** 

Assistance compiling contracts ⁪**[chconts]** 

Assistance with training ⁪ **[chtrn]** 

Assistance with recruiting ⁪**[chrecrt]** 

Assistance with interviews ⁪**[chsintr]** 

Bank of emergency staff ⁪**[chemrg]** 

Any other back-up service ⁪**[chotbup]** 

General advice and support ⁪**[chgen]** 

Not applicable **[-3]** 

f) If you choose to receive help from the **recruitment support service**, what difference does this service make to your experience of direct payments? **[sigrcs]**

Could not mange without them ⁪ **[1]** 

Could manage without them but would feel less supported ⁪ **[2]** 

Could manage without them ⁪ **[3]** 

Not applicable **[-3]** 

4.3 a) Were you offered the services from an **Employment Agency**? **[offempag]**

(Employment agencies are involved in introducing care workers to users. They often provide workers on a rota basis, each staying for two weeks at a time. Most deal with carer workers who live in. The agency charges a fee and deals with tax, National Insurance and employers liability insurance but, crucially the workers are paid directly by the older people or their carer)

Yes ⁪ **[1]** 

Not offered ⁪ **[2]** 

Not available ⁪ **[3]** 

Don’t know ⁪ **[-2]** 

b) Did you choose to receive services from an **Employment Agency**? **[chempag]**

Yes ⁪**[1]** 

No ⁪**[2]** 

Don’t know ⁪**[-2]** 

Not applicable **[-3]** 

c) If not, why not?

_______________________________________________________________________________________________________________________________________________________________________________________________________________

d) *Which services were you offered?*

### Services Offered

Care worker introduction service ⁪ **[ofcwintr]** 

Employee scheduling/ rotation service ⁪ **[ofesche]** 

Finance and insurance management service ⁪ **[offinins]** 

Not applicable **[-3]** 

e) *Which services did you choose?*

### Services Chosen

Care worker introduction service ⁪ **[chcwintr]** 

Employee scheduling/ rotation service ⁪ **[chesche]** 

Finance and insurance management service ⁪ **[chfinins]** 

Not applicable **[-3]** 

f) If you choose to receive help from an **employment agency**, what difference does this service make to your experience of direct payments? **[sigempag]**

Could not mange without them ⁪ **[1]** 

Could manage without them but would feel less supported ⁪ **[2]** 

Could manage without them ⁪ **[3]** 

Not applicable **[-3]** 

4.4 a) Were you offered the services of an **Employment Business**? **[offempbu]**

(Employment businesses contract care workers, pay them and place them to work under the direct control of the hirer)

Yes ⁪ **[1]** 

Not offered ⁪ **[2]** 

Not available ⁪ **[3]** 

Don’t know ⁪ **[-2]** 

b) Did you choose to receive services from an Employment Business? **[chemplbu]**

Yes ⁪**[1]** 

No ⁪**[2]** 

Don’t know ⁪**[-2]** 

c) If not, why not?

_________________________________________________________________________________________________________________________________________________________________________________________________________________________________________________________________________________________________________________________________________________________

d) If you choose to receive help from an employment business, what difference does this service make to your experience of direct payments? **[sigemplb]**

Could not mange without them ⁪ **[1]** 

Could manage without them but would feel less supported ⁪ **[2]** 

Could manage without them ⁪ **[3]** 

Not applicable **[-3]** 

4.5 a) Do you make any payments for services received? **[payserv]**

Yes ⁪**[1]** 

No ⁪**[2]** 

Don’t know ⁪**[-2]** 

b) If so, how much do you pay? **[ampdpsup]**

⁪ Per week/ month/ year 

Not applicable **[-3]** 

c) Do you know if this covers the full cost of the services you receive or of there is any subsidy from your local authority?

_________________________________________________________________________________________________________________________________________________________________________________________________________________________________________________________________________________________________________________________________________________________

d) How much is the amount that you pay (approximately) as a proportion of your total income for direct payments? **[percpdps]**

⁪% 

Not applicable **[-3]** 

**SECTION 5** – INFORMAL CARE

5.1 a) Do you have an informal carer, relative or friend who helps you to manage with ADLs or IADLs on a regular basis? **[hasIC]**

Yes ⁪**[1]** 

No ⁪**[2]** 

ADLs ⁪**[crhpadl]** 

IADLs **[crhpiadl]** 

Not applicable **[-3]** 

b) What is the gender of your main carer? **[gdrcr]**

Male ⁪ **[1]** 

Female **[2]** 

Don’t know **[-2]** 

Not applicable **[-3]** 

c) What is the relationship of your main carer to you? **[rlmncr]**

Son ⁪ **[1]** 

Daughter **[2]** 

Spouse (husband)/ male partner **[3]** 

Spouse (wife)/ female partner **[4]** 

Brother  **[5]** 

Sister  **[6]** 

Grandchild  **[7]** 

Neighbour/ friend  **[8]** 

Not applicable **[-3]** 

e) Does your main carer in paid employment? **[crempl]**

Yes ⁪ **[1]** 

No **[2]** 

Don’t know **[-2]** 

Not applicable **[-3]** 

d) Is your main carer paid to care for you through direct payments? **[mncrpd]**

Yes ⁪ **[1]** 

No **[2]** 

Don’t know **[-2]** 

Not applicable **[-3]** 

e) What is your main carers’ occupation?

__________________________________________________________________________________________________________________________________________

5.2 a) Does you **main** informal carer, relative or friend help you to *manage your direct payments*?

Yes ⁪**[1]** 

No ⁪**[2]** 

Don’t know ⁪**[-2]** 

Not applicable **[-3]** 

If so, what difference does their assistance make to your experience of direct payments? **[siginfcr]**

Could not mange without them ⁪ **[1]** 

Could manage without them but would feel less supported ⁪ **[2]** 

Could manage without them ⁪ **[3]** 

Not applicable **[-3]** 

c) How do you feel about the amount of support they give you?

_______________________________________________________________________________________________________________________________________________________________________________________________________________

5.3 a) If you have an informal carer who helps you, have they received much support in their role from either a local support group or people from the local authority? **[ssspinf]**

A lot of support ⁪**[1]** 

Some support ⁪**[2]** 

Little support ⁪**[3]** 

No support ⁪**[4]** 

Not applicable **[-3]** 

b) Have they had their own (carer’s) assessment of their needs? **[carass]**

Yes ⁪**[1]** 

No ⁪**[2]** 

Don’t know ⁪**[-2]** 

Not applicable **[-3]** 

c) If yes, do they now receive a care package? **[carcpck]**

Yes ⁪**[1]** 

No ⁪**[2]** 

Don’t know ⁪**[-2]** 

Not applicable **[-3]** 

d) If yes, how many hours is this per week? **[carerscp]**

Hours per week on average 

Not applicable **[-3]** 

5.3 a) Do you any other informal carer, relative or friend who helps you to manage with ADLs or IADLs on a regular basis? **[athrcr]**

Yes ⁪**[1]** 

No ⁪**[2]** 

Not applicable **[-3]** 

b) What is the relationship of your other informal carer(s) to you? **[rlothcr]**

Granddaughter **[1]** 

Grandson **[2]** 

Daughter-in-law **[3]** 

Son-in-law **[4]** 

Various **[5]** 

Son  **[6]** 

Daughter  **[7]** 

Cousin **[8]** 

Neighbour/ friend  **[9]** 

Not applicable **[-3]** 

d) Is your secondary informal carer paid to care for you through direct payments? **[athrcrpd]**

Yes ⁪ **[1]** 

No **[2]** 

Don’t know **[-2]** 

Not applicable **[-3]** 

**SECTION 6** – EMERGENCY PROVISION

6.1 Do you feel confident that you have sufficient arrangements in place should you be unwell or otherwise in the event of an emergency? **[cnfdemr]**

Yes ⁪**[1]** 

No ⁪**[2]** 

Don’t know ⁪**[-2]** 

6.2 Have you been hospitalised unexpectedly in the past twelve months? **[hosptwmn]**

Yes ⁪**[1]** 

No ⁪**[2]** 

Don’t know ⁪**[-2]** 

6.3 Do you feel more or less confident than you would if using standard social services? **[compss]**

More confident ⁪**[1]** ⁪

The same ⁪**[2]** 

Less confident ⁪**[3]** 

Don’t know ⁪**[-2]** 

**SECTION 7** – ACTIVITIES OF DAILY LIVING – BARTHEL INDEX

##### Bowels [bowels]

0 = Incontinent ⁪

1 = Occasional incontinence ⁪

2 = Continent ⁪

##### Bladder [bladder]

0 = Incontinent or catheterised/ unable to manage ⁪

1 = Occasional accident (max 1x per 24 hours) ⁪

2 = Continent (for over 7 days) ⁪

##### Grooming [grooming]

0 = Needs Help ⁪

1 = Independent (face/ hair/teeth/shaving) ⁪

##### Toilet use [toilet]

0 = Dependent ⁪

1 = Needs some help but can do something ⁪

2 = Independent (on and off. Dressing, wiping) ⁪

##### Transfer [transfer]

0 = Unable ⁪

1 = Major help ⁪

2 = Minor help ⁪

3 = Independent ⁪

##### Mobility [mobility]

0 = Immobile ⁪

1 = Wheelchair independent ⁪

2 = Walks with help of one person (verbal or physical) ⁪

3 = Independent (but may use any aid, e.g. stick) ⁪

##### Dressing [dressing]

0 = Dependent ⁪

1 = Needs help, but can do half unaided ⁪

2 = Independent ⁪

##### Stairs [stairs]

0 = Unable ⁪

1 = Needs help (verbal, physical, carrying aid) ⁪

2 = Independent up and down ⁪

##### Bathing [bathing]

0 = Dependent ⁪

1 = Independent ⁪

SCORE = ⁪ **[adl]**

**SECTION 8** – INSTRUMENTAL ACTIVITIES OF DAILY LIVING – LAWTON INDEX

**Ability to use telephone [phone]**

1 = Operates telephone on own initiative ⁪**[1]**

(Able to look up numbers, dial, receive and make calls, without help)

1 = Dials a few well-known numbers ⁪**[2]**

1 = Answers telephone but does not dial ⁪**[3]**

0 = Does not use telephone at all ⁪**[4]**

##### Shopping [shopping]

1 = Takes care of all shopping needs independently ⁪**[1]**

0 = Shops independently for small purchases ⁪**[2]**

0 = Needs to be accompanied on any shopping trip ⁪**[3]**

0 = Completely unable to shop ⁪**[4]**

**Food preparation** **[food]**

1 = Plans, prepares and serves adequate meals independently ⁪**[1]**

0 = Prepares adequate meals if supplied with ingredients ⁪**[2]**

0 = Heats serves and prepares meals, or prepares meals, ⁪**[3]**

but does not maintain adequate diet

0 = Needs to have meals prepared and served ⁪**[4]**

##### Housekeeping [house]

1 = Maintains house alone or with occasional assistance ⁪**[1]**

(e.g. heavy domestic work help)

1 = Performs light daily tasks such as dishwashing, bed making ⁪**[2]**

1 = Performs daily tasks but cannot maintain an acceptable level of cleanliness ⁪**[3]**

1 = Needs help with all home maintenance tasks ⁪**[4]**

0 = Does not participate in any housekeeping tasks ⁪**[5]**

##### Laundry [laundry]

1 = Does personal laundry completely ⁪**[1]**

1 = Launders small items; rinses stockings, etc. ⁪**[2]**

0 = All laundry must be done by others ⁪**[3]**

##### Mode of transportation [transpor]

1 = Travels independently on public transportation or drives own car ⁪**[1]**

1 = Arranges own travel via taxi, but does but otherwise use public transport ⁪**[2]**

1 = Travels on public transportation when accompanied by another ⁪**[3]**

0 = Travel limited to taxi or automobile with assistance of another ⁪**[4]**

0 = Does not travel at all ⁪**[5]**

##### Responsibility for own medications [medicat]

1 = Is responsible for taking medication in correct dosages at correct time ⁪**[1]**

0 = Takes responsibility if medication is prepared in advance in separate dosage ⁪**[2]**

0 = Is not capable of dispensing own medication ⁪**[3]**

**Ability to handle finances [finance]**

1 = Manages finances independently (budgets, writes checks, ⁪**[1]**

pays rent, bills, goes to bank), collects and keeps track of income

1 = Manages day-to-day purchases, but needs help with banking, ⁪**[2]**

major purchases etc.

0 = Incapable of handling money ⁪**[3]**

SCORE = ⁪**[iadl]**

**SECTION 9** – ASSESSMENT

9.1 How often are your needs assessed by social services? **[freqasss]**

Every ⁪ weeks/ months/ years

9.2 In your view is this frequency…? **[satfreas]**

About right ⁪ **[1]**

Too often ⁪ **[2]**

Not enough ⁪ **[3]**

9.3 a) How do you feel about these assessments? **[satasss]**

Very satisfied ⁪ **[1]**

Satisfied ⁪ **[2]**

Neutral ⁪ **[3]**

Unsatisfied ⁪ **[4]**

V. Unsatisfied ⁪ **[5]**

Don’t know ⁪ **[-2]**

b) If you are unsatisfied with the assessments why is this?

_______________________________________________________________________________________________________________________________________________________________________________________________________________________________________________________________________________________________________________________________________________________________________________________

9.4 Do you feel that the information gathered through your assessment is used by social services to improve the support that they can give you? **[assimps]**

[Prompt] Do you feel that it aids their understanding of your situation so that they are better informed when you need to contact them?

Yes ⁪**[1]**

No ⁪**[2]**

Don’t know ⁪**[-2]**

9.5 Do you feel that the assessment helps to ensure that your needs are met? **[assennm]**

Yes ⁪**[1]**

No ⁪**[2]**

Don’t know ⁪**[-2]**

9.6 a) Do you have any contact with social services in between assessments? **[cnssbt]**

Yes ⁪**[1]**

No ⁪**[2]**

Don’t know ⁪**[-2]**

b) If so, what sort of contact?

_______________________________________________________________________________________________________________________________________________________________________________________________________________________________________________________________________________________________________________________________________________________________________________________

**SECTION 10** - PURCHASING CARE

10.1 a) Do you buy all your support from direct payments? **[spbghtdp]**

Yes ⁪**[1]**

No ⁪**[2]**

b) If not, do you also use any care provided through agencies or the local council support services? **[useothr]**

Yes ⁪**[1]**

No ⁪**[2]**

10.2 a) Do you feel that the nature of care that you purchase with direct payments differ from what you would have otherwise received through social services? [prompt] For example, do you choose to receive help with different tasks and/or at different times? **[spdpdif]**

Yes ⁪**[1]**

No ⁪**[2]**

Don’t know ⁪**[-2]**

b) If yes, please give some examples…

_______________________________________________________________________________________________________________________________________________________________________________________________________________________________________________________________________________________________________________________________________________________________________________________

10.3 a) Are there services or support that you personally would like to be able to use your direct payments funds to purchase but that you are not allowed to (such as home improvements, respite)? **[serunbby]**

Yes ⁪**[1]**

No ⁪**[2]**

Don’t know ⁪**[-2]**

b) If yes, please give comments…

_______________________________________________________________________________________________________________________________________________________________________________________________________________________________________________________________________________________________________________________________________________________________________________________

10.4 a) Do you feel restricted in your choice of services because of their costs? **[reschcst]**

Yes ⁪**[1]**

No ⁪**[2]**

Don’t know ⁪**[-2]**

b) If yes, please give comments…

_______________________________________________________________________________________________________________________________________________________________________________________________________________________________________________________________________________________________________________________________________________________________________________________

c) For example, could you afford local agency services if you wished to use them? **[afffdage]**

Yes ⁪**[1]**

No ⁪**[2]**

Don’t know ⁪**[-2]**

d) Any comments…

_______________________________________________________________________________________________________________________________________________________________________________________________________________________________________________________________________________________________________________________________________________________________________________________

10.5 Have you found having funds to purchase services rather than being allocated care through social services allows you to adapt the support more flexibly to your particular needs? (in what particular ways?) **[dpmrflx]**

Yes ⁪**[1]**

No ⁪**[2]**

Don’t know ⁪**[-2]**

10.6 If you purchase services from agencies do you feel that the agency responds better to your needs since you purchase their services directly? **[agrspbtr]**

Yes ⁪**[1]**

No ⁪**[2]**

Don’t know ⁪**[-2]**

10.7 What does ‘responds better to your needs’ mean to you?

_______________________________________________________________________________________________________________________________________________________________________________________________________________________________________________________________________________________________________________________________________________________________________________________

10.8 Which (if any) of the following are the most significant to you in relation to your choice to purchase care through direct payments?

**[If more than one please rank in order of importance 1 = most important; 8 = least important; -3 = not applicable; -2 don’t know; 99 = important but unable to rank]**

**[USE PROMPT CARD]**

Level of control ⁪**[lvcont]**

Continuity of care /being able to establish a regular carer ⁪**[cntrcr]**

Quality of care ⁪**[qcr]**

Flexibility in support and care provided (e.g. timing, activities) ⁪**[flexcr]**

Choice over who provides care ⁪**[choicr]**

Opportunity to build a relationship with carer ⁪**[regcr]**

Receiving care that is more responsive to your needs ⁪**[respcr]**

10.9 Have your needs changed while using direct payments? **[ndschgd]**

Yes ⁪**[1]**

No ⁪**[2]**

Don’t know ⁪**[-2]**

b) If so, did you have any difficulty meeting your care needs, or recruiting the care that you needed as your needs changed? **[dfcnchgd]**

Yes ⁪**[1]**

No ⁪**[2]**

Please explain…

_______________________________________________________________________________________________________________________________________________________________________________________________________________________________________________________________________________________________________________________________________________________________________________________

c) How does this experience compare, to previous experiences you may have had with standard services, at times when your needs had changed?

_______________________________________________________________________________________________________________________________________________________________________________________________________________________________________________________________________________________________________________________________________________________________________________________

10.10 a) Are there any needs, which you consider could not be met through the purchase of care via direct payments? **[nnmtwdp]**

Yes ⁪**[1]**

No ⁪**[2]**

Don’t know ⁪**[-2]**

b) If yes, why do you feel this?

_______________________________________________________________________________________________________________________________________________________________________________________________________________________________________________________________________________________________________________________________________________________________________________________

10.10 a) Do you have any health needs? **[hlthnds]**

Yes ⁪**[1]**

No ⁪**[2]**

Don’t know ⁪**[-2]**

b) Do health (i.e. the PCT) contribute to the cost of your DP’s? **[hlthpy]**

Yes ⁪**[1]**

No ⁪**[2]**

Don’t know ⁪**[-2]**

c) If so, are you able to purchase health services with your direct funds? **[purhlth]**

Yes ⁪**[1]**

No ⁪**[2]**

Don’t know ⁪**[-2]**

d) If not, why not?

______________________________________________________________________________________________________________________________________________________________________________________________________________________________________________________________________________________________________________________________________________________________________________________

10.12 Are there any things that you consider limit the flexibility provided by direct payments?

[prompt] Such as: difficulty recruiting, limits to your capacity to manage direct payments, or anything else that comes to mind? **[lmtsflex]**

Yes ⁪**[1]**

No ⁪**[2]**

Don’t know ⁪**[-2]**

If yes, please give comments…

_______________________________________________________________________________________________________________________________________________________________________________________________________________________________________________________________________________________________________________________________________________________________________________________

10.13 Has your experience with direct payments changed over time? **[expchgdt]**

[i.e Has it become easier or more difficult or more or less suitable to your needs over time?]

Yes ⁪

No ⁪

Don’t know ⁪

If so, how?

_______________________________________________________________________________________________________________________________________________________________________________________________________________________________________________________________________________________________________________________________________________________________________________________

**SECTION 11**– BEING AN EMPLOYER

11.1 If you obtain care from an individual or individuals (i.e. a personal assistant or care worker) how did you recruit this person? **[hwrctpa]**

Through advertising ⁪**[1]**

Via an agency ⁪**[2]**

Via the local authority ⁪**[3]**

Via a direct payments support centre/ independent living centre ⁪**[4]**

From a list of personal assistants ⁪**[5]**

Through a friend ⁪**[6]**

Existing carer (paying privately) ⁪**[7]**

Existing carer (informal) ⁪**[8]**

Other ⁪**[9]**

11.2 Why did you choose to seek care by this method?

____________________________________________________________________________________________________________________________________________________________________________________________________________________________________________________________________________________________________________

11.3 What do you look for in an employee?

____________________________________________________________________________________________________________________________________________________________________________________________________________________________________________________________________________________________________________

11.4 What are the main qualities that you expect the people you employ to have?

____________________________________________________________________________________________________________________________________________________________________________________________________________________________________________________________________________________________________________

11.5 What are the most difficult aspects of being a boss and how do you get around these?

____________________________________________________________________________________________________________________________________________________________________________________________________________________________________________________________________________________________________________

11.6 What are the main benefits of being a boss?

_______________________________________________________________________________________________________________________________________________________________________________________________________________________________________________________________________________________________________________________________________________________________________________________

11.7 What is the average length of time that each of your personal assistants is employed by you? **[lotpaemp]**

⁪ years

11.8 How many personal assistants have you had since first starting to use direct payments?

⁪ **[npa]** in ⁪ years **[nyrs]**

11.9 Did you experience more changes (higher turnover) in personal assistants during your first six months using direct payments? **[hghtrbeg]**

Yes ⁪**[1]**

No ⁪**[2]**

Don’t know ⁪**[-2]**

11.10 Have you ever used agency services (such as home care, domiciliary care services) to fill the gap in between recruiting personal assistants? **[agingps]**

Yes ⁪**[1]**

No ⁪**[2]**

Don’t know ⁪**[-2]**

11.11 Did you have any assistance or training in how to go about recruiting someone? **[assrecrt]**

Yes ⁪**[1]**

No ⁪**[2]**

Don’t know ⁪**[-2]**

11.12 If your personal assistant(s)/ care worker(s) is not a member of an agency, does he or she work for anybody else?

**[pa1othru] [pa2othru] [pa3othru]**

Personal Assistant (1) Personal Assistant (2) Personal Assistant (3)

Yes ⁪**[1]** Yes ⁪**[1]** Yes ⁪**[1]**

No ⁪**[2]**  No ⁪**[2]** No ⁪**[2]**

Don’t know ⁪**[-2]** Don’t know ⁪**[-2]** Don’t know ⁪**[-2]**

a) If yes, does he/she have another job, which is not working for a direct payment user?

**[pa1sndjb] [pa2sndjb] [pa3sndjb]**

Personal Assistant (1) Personal Assistant (2) Personal Assistant (3)

Yes ⁪**[1]** Yes ⁪**[1]** Yes ⁪**[1]**

No ⁪**[2]**  No ⁪**[2]** No ⁪**[2]**

Don’t know ⁪**[-2]** Don’t know ⁪**[-2]** Don’t know ⁪**[-2]**

b) If so, what is this job?

_______________________________________________________________________________________________________________________________________________________________________________________________________________________________________________________________________________________________________________________________________________________________________________________

c) If no, how many other direct payment users does he/ she work for?

**[nsupa1] [nsupa2] [nsupa3]**

Personal Assistant (1) Personal Assistant (2) Personal Assistant (3)

⁪ DPU ⁪ DPU ⁪ DPU

Don’t know ⁪**[-2]** Don’t know ⁪**[-2]** Don’t know ⁪**[-2]**

11.13 What did your personal assistant(s) do before working for you?

_______________________________________________________________________________________________________________________________________________________________________________________________________________________________________________________________________________________________________________________________________________

11.14 What age range does/ do your personal assistant(s) fall under?

**[agepa1] [agepa2] [agepa3]**

Personal Assistant (1) Personal Assistant (2) Personal Assistant (3)

16-20 ⁪**[1]**  16-20 ⁪**[1]**  16-20 ⁪**[1]**

21-25 ⁪**[2]**  21-25 ⁪**[2]**  21-25 ⁪**[2]**

26-30 ⁪**[3]** 26-30 ⁪**[3]**  26-30 ⁪**[3]**

30-39 ⁪**[4]**  30-39 ⁪**[4]**  30-39 ⁪**[4]**

40-49 ⁪**[5]** 40-49 ⁪**[5]**  40-49 ⁪**[5]**

50-59 ⁪**[6]**  50-59 ⁪**[6]**  50-59 ⁪**[6]**

60-69 ⁪**[7]**  60-69 ⁪**[7]**  60-69 ⁪**[7]**

70-79 ⁪**[8]**  70-79 ⁪**[8]**  70-79 ⁪**[8]**

80+ ⁪**[9]**  80+ ⁪**[9]** 80+ ⁪**[9]**

11.15 Do any of your personal assistants have any qualifications in care?

**[qulfpa1] [qulfpa2] [qulfpa3]**

Personal Assistant (1) Personal Assistant (2) Personal Assistant (3)

Yes ⁪**[1]** Yes ⁪**[1]** Yes ⁪**[1]**

No ⁪**[2]**  No ⁪**[2]** No ⁪**[2]**

Don’t know ⁪**[-2]** Don’t know ⁪**[-2]** Don’t know ⁪**[-2]**

b) If so, what qualifications do they have?

_______________________________________________________________________________________________________________________________________________________________________________________________________________________________________________________________________________________________________________________________________________

11.16 Do you think it would be a good thing if all people that work as personal assistants outside of agencies (such as the people you employ) were required to be registered under the national regulatory framework that now covers other areas of care such as residential care and domiciliary (home) care? [This may mean that they have to do minimum training]

**[rgltngd]**

Yes ⁪**[1]**

No ⁪**[2]**

Don’t know ⁪**[-2]**

b) If not, why not?

_________________________________________________________________________________________________________________________________________________________________________________________________________________________________

______________________________________________________________________________________________________________________________________________________

11.17 Have you experienced any difficulties with the people who provide care to you, since using direct payments? **[prbwcrs]**

PROMPT: (For instance, problems with carers not turning up. Or instances in which you feel that your carers tried to take advantage of you – such as demanding further payments)

Yes ⁪**[1]**

No ⁪**[2]**

Don’t know ⁪**[-2]**

If so, what were these problems?

_______________________________________________________________________________________________________________________________________________________________________________________________________________________________________________________________________________________________________________________________________________________________________________________

11.18 Do you consider yourself to be vulnerable when purchasing care, or do you have difficulty trusting your employees?

###### Yes, feel vulnerable ⁪ [1] [vulwcrs]

###### Yes, have *difficulty* trusting employee ⁪ [1] [diftstcr]

###### Yes, *unable* to trust employee ⁪ [1] [utstcrs]

###### No, none of these ⁪[2]

b) If so, have you ever done anything about this? **[action]**

Yes ⁪**[1]**

No ⁪**[2]**

Don’t know ⁪**[-2]**

c) Is so, what have you done [i.e. Do you contact your local ILC, or your care manager?]

_______________________________________________________________________________________________________________________________________________________________________________________________________________________________________________________________________________________________________________________________________________

11.19 Do you consider that there is more ‘give and take’ between you and your care workers/ personal assistants than there would be if you were not their direct employer? **[mrgvtk]**

Yes ⁪**[1]**

No ⁪**[2]**

Don’t know ⁪**[-2]**

11.20 Other than the fact that they provide the care that you request, do any of the following explain why you have kept your personal assistant(s)/ care workers?

**[USE PROMPT CARD]**

- Mutual affection – You would feel bad letting them down, i.e. sacking them.⁪**[1]**
- Pro-social disposition – You continue to employ this person not because you care for them but because it would make you feel bad if you let them go. ⁪**[2]**
- Repeated interactions – You believe that there is the possibility of sustaining the relationship that you have with this person and you expect that this person will stay being your personal assistant/ care worker. ⁪**[3]**
- Reputation – You trust your personal assistant(s)/ care worker(s) because you have a sense of their disposition. ⁪**[4]**
- None of these ⁪**[5]**
- Don’t know ⁪**[-2]**,

If none of these apply, then please could you try to explain why you retain the care workers/ personal assistants that you employ.

________________________________________________________________________________________________________________________________________________________________________________________________________________________________________________________________________________________________________________________________

11.21 Have you provided your employee(s) with a job specification or list of duties? **[jobspec]**

Yes ⁪**[1]**

No ⁪**[2]**

Don’t know ⁪**[-2]**

b) If not, why not? **[whynjs]**

Provided by employment agency/ business ⁪**[1]**

Provided by care worker agency ⁪**[2]**

Did not think to provide one ⁪**[3]**

Did not know how to write one ⁪**[4]**

Prefer to give verbal instructions ⁪**[5]**

11.22 Have you provided your employee(s) with a contract? **[pacntrt]**

Yes ⁪**[1]**

No ⁪**[2]**

Don’t know ⁪**[-2]**

11.23 Do you provide your care workers(s) with a guarantee of a minimum level of work per month or per week? **[minlwk]**

Yes ⁪**[1]**

No ⁪**[2]**

Don’t know ⁪**[-2]**

b) If yes, please give details.

____________________________________________________________________________________________________________________________________________________________________________________________________________________________________________________________________________________________________________

11.24 Did you train your employee(s)? **[train]**

Yes ⁪**[1]**

No ⁪**[2]**

Don’t know ⁪**[-2]**

b) If so, what sort of training was required?

_______________________________________________________________________________________________________________________________________________________________________________________________________________________________________________________________________________________________________________________________________________

c) Did you receive any assistance with this? **[asswtrn]**

Yes ⁪**[1]**

No ⁪**[2]**

Don’t know ⁪**[-2]**

d) If so, from who?

_______________________________________________________________________________________________________________________________________________________________________________________________________________________________________________________________________________________________________________________________________________

11.25 Is your personal assistant(s) registered as being self-employed? **[regsemp]**

Yes ⁪**[1]**

No ⁪**[2]**

Don’t know ⁪**[-2]**

Not applicable ⁪**[-3]**

11.26 If your personal assistant(s) is not self-employed, do you deal with organising your employees’ tax and National Insurance yourself? **[sutni]**

Yes ⁪**[1]**

No ⁪**[2]**

Don’t know ⁪**[-2]**

Not applicable ⁪**[-3]**

a) If not, why not? **[whytni]**

Managed by friend ⁪**[1]**

Managed by spouse ⁪**[12**

Managed by son or daughter ⁪**[3]**

Managed by grandson or granddaughter ⁪**[4]**

Other relative ⁪**[5]**

Managed by direct payments support worker ⁪**[6]**

Managed by care manager ⁪**[7]**

Do not organise employees’ tax and National Insurance contributions ⁪**[8]**

b) If yes, how do you find complying with legal responsibilities, such as payroll, tax and National Insurance?

[Prompt: simple, easy, ok, difficult, cumbersome, time consuming]

_______________________________________________________________________________________________________________________________________________________________________________________________________________________________________________________________________________________________________________________________________________________________________________________

11.27 Do you provide a payroll slip to your employee? **[payroll]**

Yes ⁪**[1]**

No ⁪**[2]**

Don’t know ⁪**[-2]**

11.28 Have you ever been employed in a dispute with any of your employees? **[dispute]**

[Prompt: This may have been a disagreement over manual handling (lifting), or a dispute over suitable pay or hours]

Yes ⁪**[1]**

No ⁪**[2]**

Don’t know ⁪**[-2]**

Details…

_______________________________________________________________________________________________________________________________________________________________________________________________________________________________________________________________________________________________________________________________________________________________________________________

b) If yes, how did you resolve this?

_______________________________________________________________________________________________________________________________________________________________________________________________________________________________________________________________________________________________________________________________________________________________________________________

11.29 If so, have did you seek or receive professional guidance (such as from ACAS)? If yes, from which organisation? **[acas]**

Yes ⁪**[1]**

No ⁪**[2]**

Don’t know ⁪**[-2]**

Details…

_______________________________________________________________________________________________________________________________________________________________________________________________________________________________________________________________________________________________________________________________________________________________________________________

11.30 Do you have any concerns about wider legal aspects of being an employer (e.g. Maternity Rights?) **[cncrnlg]**

Yes ⁪**[1]**

No ⁪**[2]**

Don’t know ⁪**[-2]**

b) If so, what would you like further information about?

_________________________________________________________________________________________________________________________________________________________________________________________________________________________________

11.31 If you receive care from a personal assistant, do you employ that/ those persons under any particular conditions. Specifically do they live-in, do they have the use of a car, do you provide meals and do you reimburse transport costs? **[spcndts]**

Live-in ⁪**[1]**

Use of a car ⁪**[2]**

Meals provided ⁪**[3]**

Transport costs reimbursed ⁪**[4]**

No special conditions ⁪**[4]**

11.32 If so, were these conditions settled upon after negotiation between your personal assistant and you? **[cndtsng]**

Yes ⁪**[1]**

No ⁪**[2]**

Comments?

______________________________________________________________________________________________________________________________________________________

11.33*I would like to find out what forms of care and support you purchase with your direct payments, and how you arrange this care to suit your needs. For this reason, I would like you to describe the services that you purchase on an average week…*

The following chart should be filled in using the abbreviations below. Combination labels only to be used where it is impossible to distinguish between basic categories of care for the time period. T = Time/ Duration

| **Categories of Care** | **Abrv.** | **Code** |
| --- | --- | --- |
| Rehabilitation/ Specialist Counselling | RH | **[1]** |
| Therapeutic management (e.g. Occupational therapy) | TH | **[2]** |
| Home Care (provision of meals, laundry, housework, shopping) | HM | **[3]** |
| Personal Care (help with getting up/ going to bed/ bathing/ eating) | PC | **[4]** |
| Health Care (administering of medicines/ treatment/ nursing care/ speech therapy) | HE | **[5]** |
| Help with social and leisure pursuits | SL | **[6]** |
| Other (please detail) | O | **[7]** |
| Combination home care and personal care  E.g. The carer comes in the morning and supervises self-care, while also doing some cleaning. | HM/PC | **[8]** |
| Combination of health care and personal care  E.g. Assists service user to take medications when undertaking personal care tasks. Or, ensures skin integrity when providing personal care. | HE/PC | **[9]** |
| Combination of personal care and social and leisure pursuits.  E.g. Carer assists in personal care tasks as required during a period of time during the day when personal care tasks do not predominate. The majority of the time is spent talking. *The service user considers the activity to fulfil a social function as well as a personal care function.*. | PC/SL | **[10]** |
| Combination of home care and social and leisure pursuits  E.g. The service users is escorted shopping with the carer. *The service user considers the activity to fulfil a social and or leisure function as well as a home care function*. | HM/SL | **[11]** |
| Other Combination (please detail) | OC | **[12]** |
| Informal care from an unpaid carer | IC | **[13]** |
| Self-funded care | SFC | **[14]** |

11.34 Do the timings of the care that you receive vary?

A little ⁪**[1]**

A lot ⁪**[2]**

Some times constant with floating period which varies ⁪**[3]**

Almost never ⁪**[4]**

|  | Code | T | Code | T | Code | T | Code | T | Code | T | Code | T | Code | T |
| --- | --- | --- | --- | --- | --- | --- | --- | --- | --- | --- | --- | --- | --- | --- |
| *Getting up/*  *Breakfast* | **[mongubc]** | **[mongubt]** | **[tuegubc]** | **[tuegubt]** | **[wedgubc]** | **[wedgubt]** | **[thrgub]** | **[thrgubt]** | **[frgubc]** | **[frgubt]** | **[satgubc]** | **[satgubt]** | **[sungubc]** | **[sungubt]** |
| *Morning/*  *Lunchtime* | **[monmlc]** | **[monmlt]** | **[tuemlc]** | **[tuemlt]** | **[wedmlc]** | **[wedmlt]** | **[thrmlc]** | **[thrmlt]** | **[frmlc]** | **[frmlt]** | **[satmc]** | **[satmlt]** | **[sunmlc]** | **[sunmlt]** |
| *Afternoon/*  *Tea time* | **[monatc]** | **[monatt]** | **[tueatc]** | **[tueatt]** | **[wedatc]** | **[wedwtt]** | **[thratc]** | **[thratt]** | **[friatc]** | **[friatt]** | **[satatc]** | **[satatc]** | **[sunatc]** | **[sunatt]** |
| *Evening/*  *Going to bed* | **[monebdc]** | **[monebdt]** | **[tueebdc]** | **[tueebdt]** | **[wedebdc]** | **[wedebdt]** | **[threbdc]** | **[threbdt]** | **[friebdc]** | **[friebdt]** | **[satebdc]** | **[satebdt]** | **[sunebdc]** | **[sunebdt]** |
| *Night time* | **[monnc]** | **[monnt]** | **[tuenc]** | **[tuent]** | **[wednc]** | **[wednt]** | **[thrnc]** | **[thrnt]** | **[frinc]** | **[frint]** | **[satnc]** | **[satnt]** | **[sunnc]** | **[sunnt]** |

**SECTION 12** – COSTS AND ADEQUACY OF DIRECT PAYMENTS

12.1 In total, how many hours of social care a week do you buy through direct payments?

⁪ Hours on average **[hpw]**

b) Of this amount how many hours of social care a week do you buy through direct payments for the following categories?

*[Calculate after interview based on response to 11.33]*

⁪ Day **[hdypw]**

⁪ Evening **[hepw]**

⁪ Weekend **[hwdpw]**

⁪ Night **[hnpw]**

12.2 Is the number of hours of social care you buy a week through direct payments equal to the number of hours of social care to which you are assessed as being entitled? **[hbwkess]**

Yes ⁪**[1]**

No ⁪**[2]**

It depends ⁪**[3]**

Don’t know ⁪**[-2]**

b) If not, do you choose to purchase additional hours? **[puraddh]**

Yes ⁪**[1]**

No ⁪**[2]**

Don’t know ⁪**[-2]**

c) If so, why?

_______________________________________________________________________________________________________________________________________________________________________________________________________________________________________________________________________________________________________________________________________________________________________________________

12.3 What is the hourly rate for care that you are paid?

⁪ Per hour (weekdays) **[hrwdy]**

⁪ Per hour (evenings) **[hreve]**

⁪ Per hour (weekends) **[hrwkd]**

⁪ Per hour (nights) **[hrnght]**

12.4 Does the amount of money that you receive for direct payments meet all of your costs?

**[cstsmt]**

Yes ⁪**[1]**

No ⁪**[2]**

Don’t know ⁪**[-2]**

b) If not, approximately how much is this excess cost per week or per month and what are these costs?

_______________________________________________________________________________________________________________________________________________________________________________________________________________________________________________________________________________________________________________________________________________________________________________________

c) If you do have unmet or extra costs how do you fund these?

_______________________________________________________________________________________________________________________________________________________________________________________________________________________________________________________________________________________________________________________________________________________________________________________

d) Have you approached anyone at the local authority about this issue? **[mtcstsla]**

Yes ⁪**[1]**

No ⁪**[2]**

Not applicable ⁪**[-3]**

If so, what was their response?

_______________________________________________________________________________________________________________________________________________________________________________________________________________________________________________________________________________________________________________________________________________________________________________________

e) If you do have excess costs, are these for any of the following? **[rsnxcs]**

To pay a higher hourly rate for care than the LA ‘direct payment rate’ ⁪⁪**[1]**

To pay extra wage related costs such as sickness and holiday benefits ⁪⁪**[2]**

Expenditure on support and advice i.e. accountancy service ⁪⁪**[3]**

Expenditure on employers’ liability insurance ⁪⁪**[4]**

Other administrative costs ⁪⁪**[5]**

Other expenditure required, to meet your care needs ⁪⁪**[6]**

(please explain)

_______________________________________________________________________________________________________________________________________________________________________________________________________________________________________________________________________________________________________________________________________________________________________________________

12.5 How much do you pay your care worker(s)/ care assistant(s)? **[pydftrts]**

a) Do you pay different rates for different times of the day?

Yes ⁪**[1]**

No ⁪**[2]**

b) If so, what rates do you pay?

⁪ Per hour (weekdays) **[whtrtwk]**

⁪ Per hour (evenings) **[whtrtev]**

⁪ Per hour (weekends) **[whtrtwd]**

c) Do you pay you care worker(s)/ personal assistant(s) different rates of pay depending on the task that they do for you? **[dtrtdtsk]**

Yes ⁪**[1]**

No ⁪**[2]**

If so, please give details.

_______________________________________________________________________________________________________________________________________________________________________________________________________________________________________________________________________________________________________________________________________________________________________________________

12.6 How often are you paid funds? **[frqpymt]**

Weekly ⁪**[1]**

Monthly ⁪**[2]**

Quarterly ⁪**[3]**

Six Monthly ⁪**[4]**

Yearly ⁪**[5]**

12.7 Does the regularity of payment suit you? **[suitfrq]**

Yes, very well ⁪**[1]**

Yes, quite well ⁪**[2]**

Neutral ⁪**[3]**

No, not very well ⁪**[4]**

No, not at all ⁪**[5]**

Any comments?

_______________________________________________________________________________________________________________________________________________________________________________________________________________________________________________________________________________________________________________________________________________________________________________________

12.8 Do you receive any other payments towards the costs of purchasing care, such as periodic amounts designed to cover extra costs such as insurance? **[prdpymts]**

Yes ⁪**[1]**

No ⁪**[2]**

Don’t know ⁪**[-2]**

Paid via membership of Independent Living Organisation? **[ilcpyins]**

**[Yes = 1; No = 2; Don’t know = -2]**

12.9 Has the amount that you receive as a direct payment ever been cut? **[dpct]**

Yes ⁪**[1]**

No ⁪**[2]**

Don’t know ⁪**[-2]**

b) If so, do you know why this was? **[whydpc]**

Yes ⁪**[1]**

No ⁪**[2]**

Don’t know ⁪**[-2]**

12.10 Has the amount that you receive as a direct payment ever been increased? **[dpincr]**

Yes ⁪**[1]**

No ⁪**[2]**

Don’t know ⁪**[-2]**

b) If so, do you know why this was? **[wydpincr]**

Yes ⁪**[1]**

No ⁪**[2]**

Don’t know ⁪**[-2]**

[prompt] Was this on the basis of your changed needs or was this due to changes in social services policies or other reasons?

_______________________________________________________________________________________________________________________________________________________________________________________________________________________________________________________________________________________________________________________________________________________________________________________

12.11 In time, the intensity and quantity of support you require may increase. Have social services ever discussed the implications of this with you in terms of costs? **[ftfdimps]**

Yes ⁪**[1]**

No ⁪**[2]**

Don’t know ⁪**[-2]**

_______________________________________________________________________________________________________________________________________________________________________________________________________________________________________________________________________________________________________________________________________________________________________________________

12.12 How do you feel about using direct payments in the long-term?

_______________________________________________________________________________________________________________________________________________________________________________________________________________________________________________________________________________________________________________________________________________________________________________________

**SECTION 13** – HOME ENVIRONMENT

13.1 I would like to discuss with you the role that you home environment plays in helping you to maintain independence. For this reason I would like to ask about any adaptations you may have had to your home…

(Table on following page)

13.2 Of the home adaptations listed are there any that you do not currently have, but that you think would make a considerable difference to your ability to live independently at home? **[hmadps]**

Yes ⁪**[1]**

No ⁪**[2]**

Don’t know ⁪**[-2]**

b) If yes, please give details….

c) Do you think that if you did have this/ these adaptation/s that this would mean that you might no longer need to purchase care to assist you with some aspects of your daily life? **[ndsxtra]**

Yes ⁪**[1]**

No ⁪**[2]**

Don’t know ⁪**[-2]**

If so, please give details…

_______________________________________________________________________________________________________________________________________________________________________________________________________________________________________________________________________________________________________________________________________________________________________________________

133 Do you currently live in? **[domicile]**

A flat ⁪**[1]**

A maisonette ⁪**[2]**

A bungalow ⁪**[3]**

A house ⁪**[4]**

A sheltered home ⁪**[5]**

An extra-care sheltered home ⁪**[6]**

13.4 One option for people with care needs who wish to maintain independent living is extra-care sheltered housing. Have you ever visited an extra-care sheltered housing facility? **[vstextra]**

Yes ⁪**[1]**

No ⁪**[2]**

[*Extra-care sheltered housing provides one or two bedroom flats with living room, kitchen and bathroom within a complex. Features include; wheelchair access throughout; level access showers; kitchen with knee spaces beneath the hob and sink. Communal facilities include a restaurant, lounge, hobbies room, informal meeting rooms and sitting areas. There is a residents’ forum where all matters about the organisation of the complex are discussed.*

Extra-care homes are not residential care and residents have assured tenancies. Most facilities have established links to the wider community (establishing events and outreach). Many operate as a base for local services (such as home care or day care). Residency includes 24- hour emergency domiciliary care support service from an on-site team. This team also provides individual package to those who choose to purchase them. The team is comprised of registered domiciliary carers that have been contracted to provide permanent on-site care. Care aims to be flexible to fluctuations in daily need.

Further services include a daily midday meal service, which may (or may not) be purchased.. In some cases these may be paid for by funds from Supporting People.]

b) Do any aspects of extra-care sheltered housing appeal to you, and if so, why?

_______________________________________________________________________________________________________________________________________________________________________________________________________________________________________________________________________________________________________________________________________________________________________________________

c) Have you ever, considered extra-care sheltered housing as an option? **[csdxtrac]**

Yes ⁪**[1]**

No ⁪**[2]**

Don’t know ⁪**[-2]**

If so, why and if not, why not?

_______________________________________________________________________________________________________________________________________________________________________________________________________________________________________________________________________________________________________________________________________________________________________________________

| Do you have any of the following in your home? | (tick) | Is having this crucial (C), significant (S) or useful (U) to you? | Was this provided to you as “daily living equipment”? | If not, did you pay for the costs of this? | If not, was this provided to you through funds from a disabled facilities grant? |
| --- | --- | --- | --- | --- | --- |
|  |  |  |  |  |  |
| Bathroom | **[bthrm]** | **[bthrmsig]** | **[bthrmdle]** | **[bthrmoop]** | **[bthrmdfg]** |
| Level Access Shower | **[1]** |  |  |  |  |
| Walk in bath | **[2]** |  |  |  |  |
| Bath board | **[3]** |  |  |  |  |
| Bath hoist | **[4]** |  |  |  |  |
| Non-slip flooring | **[5]** |  |  |  |  |
| Grab rails | **[6]** |  |  |  |  |
| Adjustable height sink | **[7]** |  |  |  |  |
| Bidet toilet or sit on bidet system | **[8]** |  |  |  |  |
| Raised toilet seat | **[9]** |  |  |  |  |
|  |  |  |  |  |  |
| Bedroom | **[bdrm]** | **[bdrmsig]** | **[bdrmdle]** | **[bdrmoop]** | **[bdrmdfg]** |
| Bed raise blocks | **[1]** |  |  |  |  |
| Height adjustable bed | **[2]** |  |  |  |  |
| Grab rails | **[3]** |  |  |  |  |
| Commode | **[4]** |  |  |  |  |
|  |  |  |  |  |  |
| General | **[gen]** | **[gensig]** | **[gendle]** | **[genoop]** | **[gendfg]** |
| Stairlift | **[1]** |  |  |  |  |
| Elevator (in the home) | **[2]** |  |  |  |  |
| Ceiling track hoist | **[3]** |  |  |  |  |
| Portable hoist | **[4]** |  |  |  |  |
| Transfer board | **[5]** |  |  |  |  |
| Slide sheets | **[6]** |  |  |  |  |
| Wheelchair | **[7]** |  |  |  |  |
| Electric wheelchair | **[8]** |  |  |  |  |
| Zimmer frame | **[9]** |  |  |  |  |
| Grab rails | **[10]** |  |  |  |  |
|  |  |  |  |  |  |
| Kitchen | **[kit]** | **[kitsig]** | **[kitdle]** | **[kitoop]** | **[kitdfg]** |
| Pull down shelves | **[1]** |  |  |  |  |
| Adjusted hob | **[2]** |  |  |  |  |
| Knee spaces beneath sink/ hob | **[3]** |  |  |  |  |
| Adapted appliances | **[4]** |  |  |  |  |
| Meal trolley | **[5]** |  |  |  |  |
|  |  |  |  |  |  |
| Living Room | **[lgrm]** | **[lgrmsig]** | **[lgrmdle]** | **[lgrmoop]** | **[lgrmdfg]** |
| Reclining/ tilting armchair | **[1]** |  |  |  |  |
| Chair raise blocks | **[2]** |  |  |  |  |
| Grab rails | **[3]** |  |  |  |  |
|  |  |  |  |  |  |
| Environmental | **[envt]** | **[envtsig]** | **[envtdle]** | **[envtoop]** | **[envtdfg]** |
| Infralink system/ Smart House technology | **[1]** |  |  |  |  |
| Remote control (TV, video, HiFi, alarm, telephone) | **[2]** |  |  |  |  |
| Environmental control (doors, windows, curtains, locks, bed, power socket, lights, computer, page turner, talking books) | **[3]** |  |  |  |  |
| Care Alarm (such as PIPER lifeline) | **[4]** |  |  |  |  |
