# Supplementary material for: Influences of service characteristics and older people’s attributes on outcomes from direct payments
Source: BMC Geriatr. 2021 Jan 2;21:1. doi: 10.1186/s12877-020-01943-8 (PMC7777219; doi:10.1186/s12877-020-01943-8)
Supplement: Supplementary file 2 — Additional file 2. [file 12877_2020_1943_MOESM2_ESM.docx]

**Supplementary Material 2 – Measures of the independent and dependent variables using in the analysis of direct payments outcome gain (DPOG).**

**Table I: Independent variable measures**

| **Indicator** | **How measured** | **Min/ Max (n)** | **Mean (SD)** |
| --- | --- | --- | --- |
| Dependency level | Service users were categorised on the basis of itemized ADL and IADL scores (cf. Table III). Moderate dependency was used as the base category.  No dependency (1), low dependency (2), moderate dependency (3), moderate-high dependency (4), high dependency (4), highest dependency (5). | 3/6 (81) | 5 (0.998) |
| Lives Alone | yes (1), no (0) | - | - |
| Significance of DPSS employment support (critical) | yes (1), no (0) | - | - |
| Adapted IADL: medication use | Is responsible for taking medication in correct dosages at correct time (1), takes responsibility if medication is prepared in advance in separate dosage (2), is not capable of dispensing own medication (3). | 1/3 (81) | 2 (0.894) |
| Adapted IADL: handling finances | Manages finances independently (budgets, writes checks, pays rent, bills, goes to bank), collects and keeps track of income (1), manages day-to-day purchases but needs help with banking major purchases (2), incapable of handling money (3). | 1/3 (81) | 2 (0.880) |
| Adapted IADL: use of transport | Travels independently on public transportation or drives own care (1), arranges own travel via taxi, but does not otherwise use public transport (2), travels on public transportation when accompanied by another (3), travel limited to taxi or automobile with assistance of another (4), does not travel at all (5). | 1/5 (81) | 4 (1.181) |
| Adapted IADL: ability to do shopping | Takes care of shopping needs independently (1), shops independently for small purchases (2), needs to be accompanied on any shopping trip (3), completely unable to shop (4). | 1/4 (81) | 3 (0.787) |
| Adapted IADL: food preparation | Plans prepares and serves adequate meals independently (1), prepares adequate meals is supplied with ingredients, heats serves and prepares meals but dies not maintain adequate diet (3), needs to have meals prepared and served (4) | 1/4 (81) | 3 (0.923) |
| ADL score | *Bowels* - incontinent (0), occasional incontinence (1), continent (2)  *Bladder* – incontinent or catheterised/ unable to manage (0), occasional accident (max 1x 24 hrs) (1)  *Grooming* – needs help (0), independent (1)  *Toilet Use* – dependent (0), needs help but can do something (1), independent (On and off. Dressing and wiping) (2)  *Transfer* – unable (0), major help (1), minor help (2), independent (3)  *Mobility* – immobile (0), wheelchair independent (1), walks with help of one person (verbal or physical) (2), independent (but may use any aid, e.g. stick) (3)  *Dressing* – dependent (0), needs help, but can do half unaided (1), independent (2)  *Stairs* – unable (0), needs help (verbal, physical, carrying aid) (1), independent (2)  *Bathing* – dependent (0), independent (1)  Maximum score = 17 | 0/17 (81) | 9 (4.982) |
| Presence of unpaid carer | yes (1), no (0) | - | - |
| Chose and received DPSSs (general and employment support) * | yes (1), no (0) | - | - |
| Use of an agency | yes (1), no (0) | - | - |
| Length of time using direct payments | In months | 2/156 (77) | 26 (27.045) |
| Difference between package size and total care input | Package size measured as hours of SS funded care per week (DP plus any other services)  Total care input calculated as hours of SS funded care per week + hours of unpaid care per week + hours of privately funded care per week.  Difference between package size and total care input = total care input (hrs/wk) – package size (hrs/wk) | 0/126 (81) | 27 (33.223) |
| Percentage of **total care input** composed of privately-funded care | (Privately funded care (hrs/wk)/Total care input (hrs/wk))*100 | 0/53 (80) | 5 (11.369) |
| Percentage of **total care input** composed of unpaid care | (Unpaid care (hrs/wk)/Total care input (hrs/wk))*100 | 0/87 (80) | 34 (31.132) |
| Percent of care package spent on home^1^ care | (Home care (hrs/wk)/Package size (hrs/wk))*100 | 0/100 (79) | 19 (30.008) |
| Percent of care package spent on combined home care/ personal care^2^ | (Combined home care/ personal care (hrs/wk)/Package size (hrs/wk))*100 | 0/100 (79) | 29 (38.207) |
| Percent of care package spent on combined home care/ social and leisure care^3^ | (Combined home care/ social and leisure care (hrs/wk)/Package size (hrs/wk))*100 | 0/100 (79) | 2 (12.977) |
| Percent of care package spent on therapeutic management^4^ | (Therapuetic management care (hrs/wk)/Package size (hrs/wk))*100 | 0/100 (79) | 4 (16.539) |

*Includes any of the following: general advice and support; assistance with recruitment; lists of personal assistants; lists of local agencies; assistance compiling job descriptions; assistance with interviews; assistance compiling contracts; assistance with training; financial advice (budgeting); bank of emergency staff; any other back-up service.

^1^ Home Care (provision of meals, laundry, housework, shopping);

^2^ Combined home care/ personal care. For example, the carer comes in the morning and supervises self-care, while also doing some cleaning.

^3^Combined home care/ social and leisure care. For example, the service user is escorted shopping with the carer. *The service user considers the activity to fulfil a social and or leisure function as well as a home care function*.

^4^Therapuetic management. For example, occupational therapy.

**Table II: Dependent variable measure for direct payments outcome gain**

| **Indicator** | **How measured** | | **Min/ Max (n)** | **Mean (SD)** |
| --- | --- | --- | --- | --- |
| Direct payments outcome gain | Weighted index value for level of met need with service– weighted index value for level of met need without service, per outcome domain: | | 10/113 (79) | 66 (23.705) |
|  | *Level of met need* | *Level of need without service* |  |  |
| **Food & Nutrition** | No problem (13)  All needs met (13)  Low unmet needs (10)  High unmet needs (0) | No problem (13)  All needs met (13)  Low unmet needs (10)  High unmet needs (0) |  |  |
| **Personal care** | No problem (32)  All needs met (32)  Low unmet needs (15)  High unmet needs (0) | No problem (32)  All needs met (32)  Low unmet needs (15)  High unmet needs (0) |  |  |
| **Safety** | No problem (9)  All needs met (9)  Low unmet needs (4)  High unmet needs (0) | No problem (9)  All needs met (9)  Low unmet needs (4)  High unmet needs (0) |  |  |
| **Social participation** | No problem (28)  All needs met (28)  Low unmet needs (17)  High unmet needs (0) | No problem (28)  All needs met (28)  Low unmet needs (17)  High unmet needs (0) |  |  |
| **Control over daily living** | All needs met (18)  Low unmet needs (16)  High unmet needs (0) | All needs met (18)  Low unmet needs (16)  High unmet needs (0) |  |  |
| **Control over home environment** | No problem (9)  All needs met (9)  Low unmet needs (4)  High unmet needs (0) | No problem (9)  All needs met (9)  Low unmet needs (4)  High unmet needs (0) |  |  |
| **Leisure pursuits** | No problem (9)  All needs met (9)  Low unmet needs (4)  High unmet needs (0) | No problem (9)  All needs met (9)  Low unmet needs (4)  High unmet needs (0) |  |  |

Note on Table II. The outcome measure used was an adapted version of OPUS with four levels for each domain: no problem, all needs met, low unmet needs and high unmet needs. This measure has been taken over by ASCOT. ASCOT includes all seven outcomes domain included in the DP adapted OPUS, plus one extra: dignity. ASCOT also has a four level scoring mechanism but the definitions of each scoring level differ. Specifically “no problem” which referred to no preexisting needs, has been replaced by “ideal state” (a) – a state in which, “the individual´s wishes and preferences in this aspect are fully met”. While “**all** needs met” has been replaced by “no needs” (b) – defined as a state in which, “the individual has no or the type of temporary trivial needs that would be expected in this area of life of someone with no impairments”. This is a crucial difference because it offers a scenario in which needs are met adequately (b) - which can be compared to scenario a, where there are either no preexisting needs or needs a met as much as the individual could ever wish for (a). ASCOT has become to tool adopted for the Adult Social Care Outcome Framework (ASCOF) which data on adult social care users is collected nationally. For national reporting responses a, and b are combined to form, “the measure on those individuals achieving the best outcomes, identifying no or limited need” (DH 2017: 11) in this area.

Source: DH (2017) The Adult Social Care Outcomes Framework 2016/17. Handbook of Definitions. Department of Health, London.

**Table III: Dependency classification**

|  | **Description of condition** |
| --- | --- |
| **Highest dependency:**  **chair-bound and dependent in transfer** | Immobile or chair-bound. Unable to transfer without the assistant of two carers. Requires a hoist for getting in and out of bed. May be incontinent or occasionally incontinent of faeces. May wear pads at night for urinary incontinence or be catheterised. May require assistance with feeding. Unable to do any IADLs. |
| **High dependency:**  **5 PADLs, can walk <2m** | Requires assistance to wash and dress. May be incontinent of urine or catheterised. Unable to walk outside, must therefore use be pushed in a wheelchair or use a power wheelchair or scooter. Unable to prepare meals. Unable to do housework or laundry. May be wheelchair independent in the home or using other aids, such as a stick or frame. Requires help and equipment to get into a bath, or alternatively a level-access shower. |
| **Moderate-high dependency:**  **2 - 4 PADLs, can walk <10m** | Able to dress partly independently but requires assistance. Requires help and equipment to get into a bath, or alternatively a level-access shower. Unable to climb stairs. May have occasional urinary incontinence. Able to transfer independently indoors with the use of aids and grab rails. Either sleeps in the ground floor, uses a stairlift or lives on one level. Has difficulty preparing meals. Unable to carry shopping or do most housework except washing up. Unable to walk outside without considerable assistance, being pushed in a wheelchair, or using a power scooter or wheelchair. |
| **Moderate dependency:**  **1 PADL in addition to bathing** | Experiences difficulty with washing body/ lower limbs at sink. Requires help getting into bath. May have difficulty transporting food around kitchen or to other rooms. Uses mobility aid outdoors. Unable to carry shopping or do heavy housework. |
| **Low dependency** | Experiences difficulty with washing lower body or back thoroughly, or with nothing generally. Has difficulty carrying shopping and doing general housework. |

**Source:** Henderson (2006)
